# Regression Modeling of Individual-Patient Correlated Discrete Outcomes with Applications to Cancer Pain Ratings

**DOI:** 10.4236/ojs.2022.124029

**Published:** 2022-08-11

**Authors:** George J. Knafl, Salimah H. Meghani

**Affiliations:** 1 School of Nursing, University of North Carolina at Chapel Hill, Chapel Hill, USA; 2 Department of Biobehavioral Health Sciences, School of Nursing, University of Pennsylvania, Philadelphia, USA

**Keywords:** Cancer Pain Ratings, Discrete Regression, Extended Linear Mixed Modeling, Likelihood-Like Cross-Validation, Nonlinear Moderation

## Abstract

**Purpose:**

To formulate and demonstrate methods for regression modeling of probabilities and dispersions for individual-patient longitudinal outcomes taking on discrete numeric values.

**Methods:**

Three alternatives for modeling of outcome probabilities are considered. Multinomial probabilities are based on different intercepts and slopes for probabilities of different outcome values. Ordinal probabilities are based on different intercepts and the same slope for probabilities of different outcome values. Censored Poisson probabilities are based on the same intercept and slope for probabilities of different outcome values. Parameters are estimated with extended linear mixed modeling maximizing a likelihood-like function based on the multivariate normal density that accounts for within-patient correlation. Formulas are provided for gradient vectors and Hessian matrices for estimating model parameters. The likelihood-like function is also used to compute cross-validation scores for alternative models and to control an adaptive modeling process for identifying possibly nonlinear functional relationships in predictors for probabilities and dispersions. Example analyses are provided of daily pain ratings for a cancer patient over a period of 97 days.

**Results:**

The censored Poisson approach is preferable for modeling these data, and presumably other data sets of this kind, because it generates a competitive model with fewer parameters in less time than the other two approaches. The generated probabilities for this model are distinctly nonlinear in time while the dispersions are distinctly non-constant over time, demonstrating the need for adaptive modeling of such data. The analyses also address the dependence of these daily pain ratings on time and the daily numbers of pain flares. Probabilities and dispersions change differently over time for different numbers of pain flares.

**Conclusions:**

Adaptive modeling of daily pain ratings for individual cancer patients is an effective way to identify nonlinear relationships in time as well as in other predictors such as the number of pain flares.

## 1. Introduction

**Figure 1.**
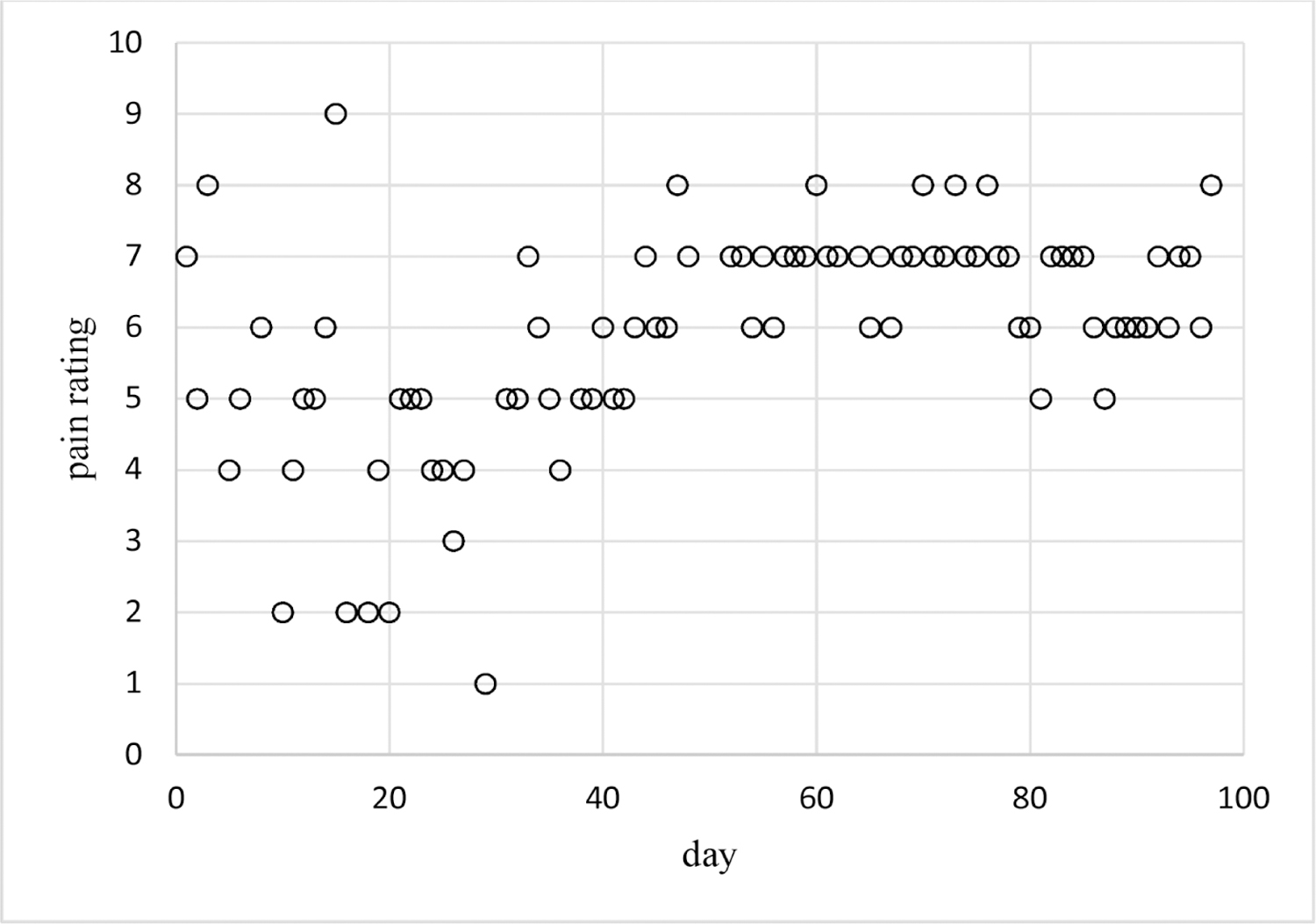
Example pain ratings over time for Cancer Patient 1.

Pain ratings are often coded as integer values from 0 – 10 with larger values indicating more pain [1] [2] [3]. These are collected by health care professionals from all kinds of patients, but are especially important for cancer patients [4]. Pain ratings collected from individual patients over multiple time points require modeling methods that account for within-patient correlation. These methods need to allow for outcomes with an arbitrary finite number of discrete numeric values since individual-patient responses can often be limited to a subset of the maximum range of 0 – 10. As an example, Figure 1 provides a plot of daily pain ratings for Cancer Patient 1. This patient provided pain ratings for 86 different days over a period of length 97 days (and so with 11 missing daily pain ratings). Observed pain ratings varied from 1 – 9 with all of these 9 ratings occurring at least one time. The plot suggests that mean pain ratings tended to increase over time with larger variability early on than later in time. Estimation of relationships like this requires regression methods for estimating probabilities, means, variances, and dispersions for observed outcome (dependent, response, *y*) values as possibly nonlinear functions of time and of other available predictors.

Generalized estimating equations (GEE) methods [5] are a possible choice for modeling such correlated pain ratings. Since pain ratings are polytomous outcomes, one could use the extensions of GEE developed by Lipsitz *et al*. [6] and Miller *et al*. [7] to handle categorical outcomes. However, these extensions involve recoding each pain rating as the vector of indicator variables for the pain rating taking on its possible values (except for one value treated as a reference category). In the case of Cancer Patient 1 with 9 possible outcome values, pain ratings at each time would be recorded as a vector of 8 indicator variables with its own 8 × 8 correlation matrix. There would be 86·85/2 = 3655 pairs of such vectors measured at different times, each of whose 8 × 8 correlation matrices would need estimation. Even for simple correlation structures like exchangeable or autoregressive, there would still be a large number 8·8 = 64 of correlation parameters. Moreover, one would need to store the overall correlation matrix of size (8·86)^2^ = 473,334 entries. Consequently, recoding correlated polytomous outcomes seems only feasible when the outcome has a small number of possible values and is measured at a small number of times. An approach is needed that treats each polytomous outcome measured at one time as univariate so that the size of the associated correlation matrix depends only on the number of measurement times and not also on the number of possible outcome values.

Likelihoods for correlated outcomes can be computationally complex except for limited cases. For this reason, Liang and Zeger [5] formulated GEE methods to avoid having to compute a likelihood by directly specifying estimating equations for mean parameters. Variances are treated as functions of the means as in generalized linear modeling [8] [9] while dispersions are treated as constant. Correlation parameters are estimated using residuals. Prentice and Zhao [10] extend the GEE estimating equations for mean parameters to also include analogous estimating equations for covariance parameters. These GEE approaches are not based on a likelihood function so that model selection criteria such as penalized likelihood criteria [11] and likelihood cross-validation scores [12] are not readily computed

Knafl and Ding [12] define a likelihood-like function *L* using the multivariate normal density computed using residuals and covariance matrices for categorical outcomes and point out that the GEE estimating equations for mean parameters correspond to differentiating the residual terms of *L* in the mean parameters while holding the covariances fixed in those parameters (see also [13]). Similar to Prentice and Zhao [10], they propose a partial extension of GEE that adds estimating equations for dispersion parameters to the GEE estimating equations for mean parameters, but they still estimate correlation parameters from residuals. Knafl and Meghani [14] consider modeling of individual-patient correlated count outcomes and compare the partial extension of GEE having some estimating equations based on differentiating *L* to extended linear mixed modeling (ELMM) based on maximizing the function *L* in all parameters including those for the means, dispersions, and correlations. ELMM generates estimating equations for all parameters as for Prentice and Zhao [10], but the estimating equations for mean parameters are not the same as for GEE (except in the special case of continuous outcomes treated as normally distributed).

Knafl and Meghani [14] compare the partial extension of GEE to ELMM for modeling individual-patient count outcomes and conclude that ELMM is preferable since it generates competitive models in less time. For that reason, only ELMM is considered here for modeling correlated discrete outcomes. They consider three correlation structures including independent correlations all equal to 0, exchangeable correlations all equal to a constant, and spatial autoregressive order 1 correlations computed as power transforms of a constant autocorrelation parameter. Exchangeable correlations are not selected in their example analyses, and so are not considered in what follows. Only spatial autoregressive order 1 (AR1) correlations based on the autocorrelation parameter *ρ* are considered in what follows. Independent correlations correspond to the special case with *ρ = 0*

Knafl and Ding [12] formulate and demonstrate an adaptive regression modeling process for identifying nonlinear relationships, controlled by likelihood cross-validation scores for comparing alternative models. These methods extend readily to modeling of discrete outcomes and are used in example analyses.

The objective of the paper is to formulate methods for analyzing discrete outcomes collected longitudinally from individual patients and to demonstrate these methods using analyses of longitudinal data on the daily pain ratings for a single cancer patient as a function of time and the number of daily pain flares. This is achieved in two parts. Section 2 addresses such methods including multinomial, ordinal, and censored Poisson probabilities as well as likelihood-like cross-validation, adaptive regression methods, and the research study whose data are used in example analyses. Section 3 presents the results of example adaptive analyses of these data including among other issues which is the preferable type of probabilities to use, how means and dispersions for the pain ratings change additively with the number of pain flares, and how the number of pain flares moderates the effect of time on means and dispersions.

## 2. Methods

Let *y*_*t(i)*_ denote discrete outcomes with a finite number of possible numeric values *v*_*u*_ for 0 ≤ *u* ≤ *K* and observed at *N* possibly non-consecutive, integer time points

t(i)∈T={t(i):1≤i≤N}

and provided by one individual patient. Let

$$p_{t(i),u}=\text{P}\left(y_{t(i)}=v_{u}\right)$$

denote associated probabilities for 0 ≤ *u* ≤ *K* and t(i)∈T. The means of these discrete outcomes satisfy

$$\mu_{t(i)}=\text{E}y_{t(i)}=\sum_{u=0}^{K}v_{u}\cdot p_{t(i),u}$$

and the variances satisfy

$$Var\left(y_{t(i)}\right)=\sum_{u=0}^{K}{\left(v_{u}-\mu_{t(i)}\right)}^{2}\cdot p_{t(i),u}=\left(\sum_{u=0}^{K}v_{u}^{2}\cdot p_{t(i),u}\right)-\mu_{t(i)}^{2}=\text{E}y_{t(i)}^{2}-\mu_{t(i)}^{2}.$$

These variances are not a direct function *V*(*μ*_*t(i)*_) of the means *μ*_*t(i)*_ as in generalized linear modeling [8] [9], but they are similar since they can be considered a function *V*(***P***_*t(i)*_) of the (*K* + 1)×1 vector ***P***_*t(i)*_ of probabilities *P*_*t(i),u*_ for the outcome *y*_*t(i)*_. Define the residuals as *e*_*t(i) =*_ *y*_*t(i) −*_ *μ*_*t(i)*_. Combine the outcomes *y*_*t(i)*_, the means *μ*_*t(i)*_, and the residuals *e*_*t(i)*_ into the *N* × 1 vectors ***y***, ***μ***, and ***e*** = ***y*** − ***μ***, respectively.

Let *x*_*t(i),j*_ denote predictor values over times t(i)∈T and over predictors indexed by 1≤j≤J for use in modeling probabilities. Combine these into the *J* × 1 vectors *x*_*t(i)*_ with transposes denoted by $x_{t(i)}^{\text{T}}$ for t(i)∈T. As formulated later, these are combined with a column vector ***β*** of coefficient parameters to estimate probabilities. Three alternatives are considered including multinomial probabilities (Section 2.1), ordinal probabilities (Section 2.2), and censored Poisson probabilities (Section 2.3). The size of the parameter vector ***β*** varies for these three alternatives.

Let $x_{t(i),j}^{\prime }$ denote predictor values over times t(i)∈T and over predictors indexed by $1\leq j\leq J^{\prime }$ for predicting dispersions. Combine these into the $J^{\prime }\times 1$ vectors $x_{t(i)}^{\prime }$ for t(i)∈T. Let $\beta^{\prime }$ denote the associated $J^{\prime }\times 1$ vector of coefficient parameters. Let $\varphi_{t(i)}$ denote dispersion values over times t(i)∈T satisfying

$$\log\varphi_{t(i)}=x_{t(i)}^{\prime \text{T}}\cdot \beta^{\prime }$$

When $x_{t(i),1}^{\prime }=1$ for t(i)∈T, the first entry $\beta_{1}^{\prime }$ of $\beta^{\prime }$ is an intercept parameter. The constant dispersion model corresponds to $x_{t(i),1}^{\prime }=1$ for t(i)∈T with $J^{\prime }=1$ Define the extended variances as

$$\sigma_{t(i)}^{2}=\varphi_{t(i)}\cdot Var\left(y_{t(i)}\right)$$

and the extended standard deviations as

$$\sigma_{t(i)}={\left(\varphi_{t(i)}\cdot Var\left(y_{t(i)}\right)\right)}^{1/2}$$

for t(i)∈T. These generate standardized residuals

$$stde_{t(i)}=e_{t(i)}/\sigma_{t(i)}$$

for t(i)∈T. Combine the extended standard deviations and the standardized residuals into the *N* × 1 vectors ***σ*** and ***stde*** = ***e/σ***, respectively.

Let ***R*** *(ρ)* denote the *N* × *N* AR1 correlation matrix for the vector ***y***. The diagonal entries of ***R*** *(ρ)* are all equal to 1 while the off-diagonal entries satisfy

$$r_{t(i),t\left(i^{\prime }\right)}=\rho^{\left|t(i)-t\left(i^{\prime }\right)\right|}$$

where $\left|t(i)-t\left(i^{\prime }\right)\right|$ denotes the absolute value of the difference $t(i)-t\left(i^{\prime }\right)$ for $t(i),t\left(i^{\prime }\right)\in T$ with $1\leq i\neq i^{\prime }\leq N$. The entries $r_{t(i),t\left(i^{\prime }\right)}$ are well-defined for −1 < *ρ* < 1 because *t*(*i*) have been assumed to be integers. These are spatial AR1 correlations that account for actual distance between observed times as opposed to non-spatial AR1 correlations with *t*(*i*) = *i* for 1 ≤ *i* ≤ *N* as usually used in GEE implementations. The *N* × *N* covariance matrix **Ʃ** for the vector ***σ*** satisfies

Σ=DIAG(σ)⋅R(ρ)⋅DIAG(σ)

where DIAG(σ) denotes the *N* × *N* diagonal matrix with diagonal entries $\sigma_{t(i)}$.

Let

$$\theta =\left(\begin{array}{l} \beta \\ \beta^{\prime }\\ \rho \end{array}\right)$$

be the column vector of the probability, dispersion, and correlation parameters. Use the multivariate normal likelihood to define the likelihood-like function *L*(*T*;***θ****)* satisfying

$$\ell (T;\theta )=\log L(T;\theta )=-e^{\text{T}}\cdot \Sigma^{-1}\cdot e/2-(\log|\Sigma |)/2-(N\cdot \log(2\cdot \pi ))/2$$

where |Σ| is the determinant of the covariance matrix **Ʃ**. Note that

$$\log|\Sigma |=\log|R(\rho )|+\sum_{i=1}^{N}\log\varphi_{t(i)}+\sum_{i=1}^{N}\log\text{Var}\left(y_{t(i)}\right),\;\varphi_{t(i)}=\exp\left(x_{t(i)}^{\prime \text{T}}\cdot \beta^{\prime }\right)$$

and

$$e^{\text{T}}\cdot \Sigma^{-1}\cdot e=\mathrm{std}e^{\text{T}}\cdot R^{-1}(\rho )\cdot \mathrm{stde}.$$

This formulation has been restricted to address data for each individual patient taking a person-centered approach to modeling longitudinal data [15] [16], which is possible because substantial amounts of outcome measurements are available for each patient. This formulation readily generalizes to handle the combined longitudinal data for multiple patients as considered in the GEE context. In that case, as pointed out by Knafl and Ding [12], the GEE estimating equations can be generated by differentiating the residual terms of ℓ(T;θ) while holding the covariance matrix terms fixed. This motivates using the ELMM approach for estimating ***θ*** based on estimating equations generated by maximizing ℓ(T;θ). Moreover, the likelihood-like function L(T;θ) can be used to generate model selection criteria. Pan [17] has formulated the quasi-likelihood information criterion (QIC) for GEE model selection. However, the QIC score does not fully account for the correlation structure while model selection criteria based on L(T;θ) fully account for the correlation structure.

The likelihood-like function L(T;θ) can be maximized to generate estimates ***θ****(T)* by solving for a zero gradient, that is,

$$g(\theta )=\frac{\partial \ell (T;\theta )}{\partial \theta }=\left(\begin{array}{l} g(\beta )\\ g\left(\beta^{\prime }\right)\\ g(\rho ) \end{array}\right)=0$$

where **0** is the zero vector,

$$g(\beta )=\frac{\partial \ell (T;\theta )}{\partial \beta }$$

is the partial derivative vector for the probability parameters,

$$g(\beta^{\prime })=\frac{\partial \ell (T;\theta )}{\partial \beta^{\prime }}$$

is the partial derivative vector for the dispersion parameters, and

$$g(\rho )=\frac{\partial \ell (T;\theta )}{\partial \rho }$$

is the partial derivative for the correlation parameter. The Hessian matrix ***H***(***θ***) has nine component submatrices:

$$H(\beta )=\frac{\partial g(\beta )}{\partial \beta }$$

for the probability parameters,

$$H(\beta^{\prime })=\frac{\partial g(\beta^{\prime })}{\partial \beta^{\prime }}$$

for the dispersion parameters,

$$H(\rho )=\frac{\partial g(\rho )}{\partial \rho }$$

for the correlation parameter,

$$H\left(\beta ,\beta^{\prime }\right)=\frac{\partial g(\beta )}{\partial \beta^{\prime }}$$

and its transpose $H\left(\beta^{\prime },\beta \right)=H^{\text{T}}\left(\beta ,\beta^{\prime }\right)$,

$$H\left(\beta ,\rho \right)=\frac{\partial g(\beta )}{\partial \rho }$$

and its transpose $H\left(\rho ,\beta \right)=H^{\text{T}}\left(\beta ,\rho \right)$, and

$$H\left(\beta^{\prime },\rho \right)=\frac{\partial g(\beta^{\prime })}{\partial \rho }$$

and its transpose $H\left(\rho ,\beta^{\prime }\right)=H^{\text{T}}\left(\beta^{\prime },\rho \right)$. Iteratively solve g(θ)=0 using Newton’s method, that is, given the current value ***θ***_*s*_ for ***θ***, the next value is given by

$$\theta_{s+1}=\theta_{s}-H^{-1}\left(\theta_{s}\right)\cdot g\left(\theta_{s}\right)$$

The solution to the estimating equations for observations indexed by *T* is denoted as

$$\theta \left(T\right)=\left(\begin{array}{l} \beta \left(T\right)\\ \beta^{\prime }\left(T\right)\\ \rho \left(T\right) \end{array}\right).$$

The partial derivative vector $g\left(\beta \right)$ varies with the probability type as do H(β), $H(\beta ,\beta^{\prime })$ and H(β,ρ). Formulas are provided for these quantities in Sections 2.1–2.3 for multinomial probabilities, ordinal probabilities, and censored Poisson probabilities, respectively. Formulas for partial derivatives common to all three probability types are provided in what follows. Details on computation of derivatives are not provided for brevity; they are available on request from the first author.

The partial derivative vector $g\left(\beta^{\prime }\right)$ has $J^{\prime }$ entries satisfying

$$g_{j}\left(\beta^{\prime }\right)={\mathrm{stdex}}_{j}^{\prime \text{T}}\cdot R^{-1}(\rho )\cdot \mathrm{stde}-\sum_{i=1}^{N}x_{t(i),j}/2$$

where ${\mathrm{stdex}}_{j}^{\prime }$ is the *N* × 1 vector with entries

$$stdex_{t(i),j}^{\prime }=x_{t(i),j}\cdot stde_{t(i)}/2$$

for $1\leq j\leq J^{\prime }$ and t(i)∈T. The derivative g(ρ) satisfies

$$g(\rho )=-{\mathrm{stde}}^{\text{T}}\cdot \frac{\partial R^{-1}(\rho )}{\partial \rho }\cdot \mathrm{stde}/2-\frac{\partial \log|R(\rho )|}{\partial \rho }/2$$

where

$$\frac{\partial R^{-1}(\rho )}{\partial \rho }=-R^{-1}(\rho )\cdot \frac{\partial R(\rho )}{\partial \rho }\cdot R^{-1}(\rho ),\;\frac{\partial \log|R(\rho )|}{\partial \rho }=trace\left(R^{-1}(\rho )\cdot \frac{\partial R(\rho )}{\partial \rho }\right).$$

For spatial AR1 correlations, $\frac{\partial R(\rho )}{\partial \rho }$ is the *N* × *N* matrix with diagonal entries all equal to 0 and off-diagonal entries equaling

$$\frac{\partial r_{t(i),t\left(i^{\prime }\right)}}{\partial \rho }=\left|t(i)-t\left(i^{\prime }\right)\right|\cdot \rho^{\left|t(i)-t\left(i^{\prime }\right)\right|-1}$$

for $1\leq i\neq i^{\prime }\leq N$.

$H(\beta^{\prime })$ has entries

$$H_{j,j}\left(\beta^{\prime }\right)=-{\mathrm{stdexx}}_{j,j^{\prime }}^{\prime \prime \text{T}}\cdot R^{-1}(\rho )\cdot \mathrm{stde}-{\mathrm{stdex}}_{j}^{\prime \text{T}}\cdot R^{-1}(\rho )\cdot {\mathrm{stdex}}_{j}^{\prime }$$

where ${\mathrm{stdexx}}_{j,j^{\prime }}^{\prime \prime }$ is the *N* × 1 vector with entries

$$\mathrm{stde}xx_{t(i),j,j^{\prime }}^{\prime \prime }=x_{t(i),j}^{\prime }\cdot x_{t(i),j^{\prime }}^{\prime }\cdot stde_{t(i)}/4$$

for $1\leq j,j^{\prime }\leq J^{\prime }$ and t(i)∈T. H(ρ) satisfies

$$H(\rho )=-{\mathrm{stde}}^{\text{T}}\cdot \frac{\partial^{2}R^{-1}(\rho )}{\partial \rho^{2}}\cdot \mathrm{stde}/2-\frac{\partial^{2}\log|R(\rho )|}{\partial \rho^{2}}/2$$

where

$$\frac{\partial^{2}R^{-1}(\rho )}{\partial \rho^{2}}=2\cdot R^{-1}(\rho )\cdot \frac{\partial R(\rho )}{\partial \rho }\cdot R^{-1}(\rho )\cdot \frac{\partial R(\rho )}{\partial \rho }\cdot R^{-1}(\rho )\;-R^{-1}(\rho )\cdot \frac{\partial^{2}R(\rho )}{\partial \rho^{2}}\cdot R^{-1}(\rho )$$

$$\frac{\partial^{2}\log|R(\rho )|}{\partial \rho^{2}}=-trace\left(R^{-1}(\rho )\cdot \frac{\partial R(\rho )}{\partial \rho }\cdot R^{-1}(\rho )\cdot \frac{\partial R(\rho )}{\partial \rho }\right)\;+trace\left(R^{-1}(\rho )\cdot \frac{\partial^{2}R(\rho )}{\partial \rho^{2}}\right).$$

Formulas for first and second derivatives of $R^{-1}(\rho )$ and log|R(ρ)| are adapted from formulas in [18]. For spatial AR1 correlations, $\frac{\partial^{2}R(\rho )}{\partial \rho^{2}}$ is the *N*×*N* matrix with diagonal entries all equal to 0 and off-diagonal entries

$$\frac{\partial^{2}r_{t(i))\cdot \left(i^{\prime }\right)}}{\partial \rho^{2}}=\left(\left|t(i)-t\left(i^{\prime }\right)\right|-1\right)\cdot \left|t(i)-t\left(i^{\prime }\right)\right|\cdot \rho^{\left|t(i)-t\left(i^{\prime }\right)\right|-2}$$

for $1\leq i\neq i^{\prime }\leq N$. $H(\beta^{\prime },\rho )$ has entries

$$H_{j}\left(\beta^{\prime },\rho \right)=\;\mathrm{stdex}{\;}_{j}^{\prime \text{T}}\cdot \frac{\partial R^{-1}(\rho )}{\partial \rho }\cdot \;\mathrm{stde}\;$$

for $1\leq j\leq J^{\prime }$.

The covariance matrix for the estimate θ(T) satisfies

$$\Sigma (\theta (T))=-H^{-1}(\theta (T)).$$

Square roots of the diagonal entries of Σ(θ(T)) can be used to generate *z* tests of zero individual model parameters. These are useful for fixed models of theoretical importance. However, these tests for parameters of adaptively generated models are usually significant as a consequence of the model selection process, and so are not reported in example analyses of Section 3.

The likelihood-like function L(T;θ) can be used to compute likelihood-like cross-validation (LCV) scores (Section 2.4) for evaluating and comparing alternative models. These scores can be used to control the adaptive modeling process (Section 2.5) for identifying power transforms of the probability predictors and of the dispersion predictors for use in nonlinear modeling of discrete outcomes.

### 2.1. Multinomial Probabilities

The probabilities $p_{t(i),u}$ are modeled multinomially using generalized logits with the smallest value *v*_0_ as the reference category (but any other value can be used instead), that is,

$$h\left(p_{t(i),u}\right)=\log\frac{p_{t(i),u}}{p_{t(i),0}}=x_{t(i)}^{\text{T}}\cdot \beta_{u}$$

for *K J* × 1 vectors ***β***_*u*_ of coefficient parameters $\beta_{u,j}$ for 1 ≤ *u* ≤ *K* and 1 ≤ *j* ≤ *J*. Combine the vectors ***β***_*u*_ over 1 ≤ *u* ≤ *K* into the composite (K⋅J)×1 vector ***β***. Altogether, there are *K*·*J* coefficient parameters for modeling the probabilities. Setting $x_{t(i),1}=1$ for t(i)∈T generates *K* intercept parameters. For t(i)∈T, the multinomial probabilities satisfy

$$p_{t(i),u}=\frac{\exp\left(x_{t(i)}^{\text{T}}\cdot \beta_{u}\right)}{1+\sum_{u^{\prime }=1}^{K}\exp\left(x_{t(i)}^{\text{T}}\cdot \beta_{u^{\prime }}\right)}$$

for 1 ≤ *u* ≤ *K* and

$$p_{t(i),0}=\frac{1}{1+\sum_{u^{\prime }=1}^{K}\exp\left(x_{t(i)}^{\text{T}}\cdot \beta_{u^{\prime }}\right)}.$$

Their partial derivatives $\frac{\partial p_{t(i),u}}{\partial \beta_{w,j}}$ satisfy

$$\frac{\partial p_{t(i),u}}{\partial \beta_{w,j}}=x_{t(i),j}\cdot p_{t(i),w}\cdot \left(1-p_{t(i),w}\right),w=u,\;\frac{\partial p_{t(i),u}}{\partial \beta_{w,j}}=-x_{t(i),j}\cdot p_{t(i),u}\cdot p_{t(i),w},w\neq u,$$

and

$$\frac{\partial p_{t(i),0}}{\partial \beta_{w,j}}=-x_{t(i),j}\cdot p_{t(i),0}\cdot p_{t(i),w},$$

for 1 ≤ *u*, *w* ≤ *K*, 1 ≤ *j* ≤ *J*, and t(i)∈T.

The derivative vector ***g***(***β***) has *K*·*J* entries $g_{w,j}(\beta )$ for 1 ≤ *w* ≤ *K* and 1 ≤ *j* ≤ *J* satisfying

$$g_{w,j}(\beta )={\mathrm{stdex}}_{w,j}^{\text{T}}\cdot R^{-1}(\rho )\cdot \mathrm{stde}-\sum_{i=1}^{N}W_{t(i),w,j}/2$$

where

$$W_{t(i),w,j}=\frac{\frac{\partial \text{Var}\left(y_{t(i)}\right)}{\partial \beta_{w,j}}}{\text{Var}\left(y_{t(i)}\right)},$$

$$\frac{\partial \text{Var}\left(y_{t(i)}\right)}{\partial \beta_{w,j}}=x_{t(i),j}\cdot p_{t(i),w}\cdot \left(v_{w}^{2}-\text{E}y_{t(i)}^{2}-2\cdot \mu_{t(i)}\cdot \left(v_{w}-\mu_{t(i)}\right)\right),$$

and ***stdex***_*w*,*j*_ is the *N* × 1 vector with entries

$$\text{stde}x_{t(i),w,j}=\frac{\partial \mu_{t(i)}}{\partial \beta_{w,j}}/\sigma_{t(i)}+stde_{t(i)}\cdot W_{t(i),w,j}/2,$$

$$\frac{\partial \mu_{t(i)}}{\partial \beta_{w,j}}=x_{t(i),j}\cdot p_{t(i),w}\cdot \left(v_{w}-\mu_{t(i)}\right),$$

for 1 ≤ *w* ≤ *K*, 1 ≤ *j* ≤ *J*, and t(i)∈T.

***H***(***β***) has entries

$$H_{w,j,w^{\prime },j^{\prime }}(\beta )=-\mathrm{stde}xx_{w,j,w^{\prime },j^{\prime }}^{\text{T}}\cdot R^{-1}(\rho )\cdot \mathrm{stde}-{\mathrm{stdex}}_{w,j}^{\text{T}}\cdot R^{-1}(\rho )\cdot {\mathrm{stdex}}_{w^{\prime },j^{\prime }}\;\;\;\;\;\;\;\;\;\;\;\;\;\;\;\;\;\;\;\;\;\;\;\;\;\;\;\;\;\;-\sum_{i=1}^{N}W_{t(i),w,j,w^{\prime },j^{\prime }}/2$$

where

$$W_{t(i),w,j,w^{\prime },j^{\prime }}=\frac{\partial W_{t(i),w,j}}{\partial \beta_{w^{\prime },j^{\prime }}}=\frac{\frac{\partial^{2}\text{Var}\left(y_{t(i)}\right)}{\partial \beta_{w^{\prime },j^{\prime }}\partial \beta_{w,j}}}{\text{Var}\left(y_{t(i)}\right)}-\frac{\frac{\partial \text{Var}\left(y_{t(i)}\right)}{\partial \beta_{w^{\prime },j^{\prime }}}\cdot \frac{\partial \text{Var}\left(y_{t(i)}\right)}{\partial \beta_{w,j}}}{{\text{Var}}^{2}\left(y_{t(i)}\right)},$$

$$\frac{\partial^{2}\text{Var}\left(y_{t(i)}\right)}{\partial \beta_{w^{\prime },j^{\prime }}\partial \beta_{w,j}}\;=x_{t(i),j}\cdot x_{t(i),j^{\prime }}\cdot p_{t(i),w}\cdot \left(1-p_{t(i),w}\right)\cdot \left(v_{w}^{2}-\text{E}y_{t(i)}^{2}-2\cdot \mu_{t(i)}\cdot \left(v_{w}-\mu_{t(i)}\right)\right)\;\;\;\;+x_{t(i),j}\cdot x_{t(i),j^{\prime }}\cdot p_{t(i),w}^{2}\cdot \left(\text{E}y_{t(i)}^{2}-v_{w}^{2}+2\cdot \left(2\cdot \mu_{t(i)}-v_{w}\right)\cdot \left(v_{w}-\mu_{t(i)}\right)\right),w^{\prime }=w,$$

$$\frac{\partial^{2}\text{Var}\left(y_{t(i)}\right)}{\partial \beta_{w^{\prime },j^{\prime }}\partial \beta_{w,j}}\;=-x_{t(i),j}\cdot x_{t(i),j^{\prime }}\cdot p_{t(i),w}\cdot p_{t(i),w^{\prime }}\cdot \left(v_{w}^{2}-\text{E}y_{t(i)}^{2}-2\cdot \mu_{t(i)}\cdot \left(v_{w}-\mu_{t(i)}\right)\right)+x_{t(i),j}\;\;\;\;\;\;\cdot x_{t(i),j^{\prime }}\cdot p_{t(i),w}\cdot p_{t(i),w^{\prime }}\cdot \left(\text{E}y_{t(i)}^{2}-v_{w^{\prime }}^{2}+2\cdot \left(2\cdot \mu_{t(i)}-v_{w^{\prime }}\right)\cdot \left(v_{w^{\prime }}-\mu_{t(i)}\right)\right),w^{\prime }\neq w,$$

while ${\mathrm{stdexx}}_{w,j,w^{\prime },j^{\prime }}$ is the *N* × 1 vector with entries

$$\text{stde}xx_{t(i),w,j,w^{\prime },j^{\prime }}=-\frac{\frac{\partial^{2}\mu_{t(i)}}{\partial \beta_{w^{\prime },j^{\prime }}\beta_{w,j}}}{\sigma_{t(i)}}+\frac{\partial \mu_{t(i)}}{\partial \beta_{w,j}}\cdot \frac{W_{t(i),w^{\prime },j^{\prime }}}{2\cdot \sigma_{t(i)}}+\text{stde}x_{t(i),w^{\prime },j^{\prime }}\cdot \frac{W_{t(i),w,j}}{2}\;\;\;\;\;\;\;\;\;\;\;\;\;\;\;\;\;\;\;\;\;\;\;\;\;\;\;\;\;\;\;\;\;\;\;\;\;-stde_{t(i)}\cdot \frac{W_{t(i),w,j,w^{\prime },j^{\prime }}}{2},$$

$$\frac{\partial^{2}\mu_{t(i)}}{\partial \beta_{w^{\prime },j^{\prime }}\partial \beta_{w,j}}=x_{t(i),j}\cdot x_{t(i),j^{\prime }}\cdot p_{t(i),w}\cdot \left(1-2\cdot p_{t(i),w}\right)\cdot \left(v_{w}-\mu_{t(i)}\right),w^{\prime }=w,$$

$$\frac{\partial^{2}\mu_{t(i)}}{\partial \beta_{w^{\prime },j^{\prime }}\partial \beta_{w,j}}=-x_{t(i),j}\cdot x_{t(i),j^{\prime }}\cdot p_{t(i),w}\cdot p_{t(i),w^{\prime }}\cdot \left(v_{w}+v_{w^{\prime }}-2\cdot \mu_{t(i)}\right),w^{\prime }\neq w,$$

for $1\leq w,w^{\prime }\leq K$, $1\leq j,j^{\prime }\leq J$, and t(i)∈T. $H(\beta ,\beta^{\prime })$ has entries

$$H_{w,j,j^{\prime }}\left(\beta ,\beta^{\prime }\right)=-{\mathrm{stdexx}}_{w,j,j^{\prime }}^{\prime \text{T}}\cdot R^{-1}(\rho )\cdot \mathrm{stde}-{\mathrm{stdex}}_{w,j}^{\text{T}}\cdot R^{-1}(\rho )\cdot {\mathrm{stdex}}_{j^{\prime }}^{\prime }$$

where ${\mathrm{stdexx}}_{w,j,j^{\prime }}^{\prime }$ is the *N* × 1 vector with entries

$${\text{stdexx}}_{t(i),w,j,j^{\prime }}^{\prime }={\text{stdex}}_{t(i),w,j}\cdot x_{t(i),j^{\prime }}^{\prime }/2$$

for 1≤j≤J, $1\leq j^{\prime }\leq J^{\prime }$, and t(i)∈T. H(β,ρ) has entries

$$H_{w,j}(\beta ,\rho )={\mathrm{stdex}}_{w,j}^{\text{T}}\cdot \frac{\partial R^{-1}(\rho )}{\partial \rho }\cdot \mathrm{stde}\;$$

for 1 ≤ *w* ≤ *K* and 1 ≤ *j* ≤ *J*.

### 2.2. Ordinal Probabilities

For t(i)∈T, define cumulative probabilities

$$p_{t(i)\leq u}=\text{P}\left(y_{t(i)}\leq v_{u}\right),0\leq u<K,$$

$$p_{t(i),\leq K}=\text{P}\left(y_{t(i)}\leq v_{K}\right)=1$$

where the values *v*_*u*_ are assumed to be in increasing order for 0 ≤ *u* ≤ *K*. The link function is cumulative logits with logits computed for lower sets of values relative to higher sets of values (but this can be reversed). Formally, for predictor values $x_{t(i),j}$, 1 ≤ *j* ≤ *J*, the cumulative probabilities $p_{t(i),\leq u}$ for 0 ≤ *u* ≤ *K* and t(i)∈T are modeled ordinally as

$$h\left(p_{t(i),\leq u}\right)=logit\left(p_{t(i),\leq u}\right)=\log\frac{p_{t(i),\leq u}}{1-p_{t(i),\leq u}}=\alpha_{u}+x_{t(i)}^{\text{T}}\cdot \beta_{K}$$

for *K* intercept parameters *α*_*u*_ and a single *J* × 1 vector ***β***_*K*_ of slope parameters ***β***_*K,j*_ for 1 ≤ *j* ≤ *J*. Combine the intercept parameters *α*_*u*_ over 0 ≤ *u* ≤ *K* into the *K* × 1 vector ***α***. Altogether, there are *K* + *J* coefficient parameters for modeling the probabilities, which are combined over 0 ≤ *u* ≤ *K* and 1 ≤ *j* ≤ *J* into the (*K* + *J*)×1 vector

$$\beta =\left(\begin{array}{c} \alpha \;\beta_{K} \end{array}\right).$$

A zero-intercept model corresponds to setting *α*_0_ = 0, but *αu* for 0 ≤ *u* ≤ *K* are nonzero. The cumulative probabilities satisfy

$$p_{t(i),\leq u}=\frac{\exp\left(\alpha_{u}+x_{t(i)}^{\text{T}}\cdot \beta_{K}\right)}{1+\exp\left(\alpha_{u}+x_{t(i)}^{\text{T}}\cdot \beta_{K}\right)}$$

for 0 ≤ *u* ≤ *K* and t(i)∈T. The cumulative probabilities are differenced to compute probabilities

$$p_{t(i),u}=\text{P}\left(y_{t(i)}=v_{u}\right)$$

that is, for t(i)∈T, define $p_{t(i),\leq -1}=0$ and then

$$p_{t(i),u}=p_{t(i),\leq u}-p_{t(i),\leq u-1}$$

for 0 ≤ *u* ≤ *K*. For 0 ≤ *u*, *w* < *K*, 1 ≤ *j* ≤ *J*, and t(i)∈T, the partial derivatives of $p_{t(i),u}$ satisfy

$$\frac{\partial p_{t(i),\leq u}}{\partial \alpha_{w}}=p_{t(i),\leq w}\cdot \left(1-p_{t(i),\leq w}\right),w=u$$

$$\frac{\partial p_{t(i),\leq u}}{\partial \alpha_{w}}=0,w\neq u,$$

$$\frac{\partial p_{t(i),\leq u}}{\partial \beta_{K,j}}=x_{t(i),j}\cdot p_{t(i),\leq u}\cdot \left(1-p_{t(i),\leq u}\right)$$

The derivative vector ***g***(***β***) has *K* + *J* entries *g*_*w*_(***β***) for 0 ≤ *w* ≤ *K* and $g_{K,j}(\beta )$ for 1 ≤ *j* ≤ *J* satisfying

$$g_{w}(\beta )={\mathrm{stdex}}_{w}^{\text{T}}\cdot R^{-1}(\rho )\cdot \mathrm{stde}-\sum_{i=1}^{N}W_{t(i),w}/2$$

$$g_{K,j}(\beta )={\mathrm{stdex}}_{K,j}^{\text{T}}\cdot R^{-1}(\rho )\cdot \mathrm{stde}-\sum_{i=1}^{N}W_{t(i),K,j}/2$$

where

$$W_{t(i),w}=\frac{\frac{\partial \text{Var}\left(y_{t(i)}\right)}{\partial \alpha_{w}}}{\text{Var}\left(y_{t(i)}\right)},$$

$$\frac{\partial \text{Var}\left(y_{t(i)}\right)}{\partial \alpha_{w}}=p_{t(i),\leq w}\cdot \left(1-p_{t(i),\leq w}\right)\cdot \left(v_{w}-v_{w+1}\right)\cdot \left(v_{w}+v_{w+1}-2\cdot \mu_{t(i)}\right),$$

$$W_{t(i),K,j}=\frac{\frac{\partial \text{Var}\left(y_{t(i)}\right)}{\partial \beta_{K,j}}}{\text{Var}\left(y_{t(i)}\right)},$$

$$\frac{\partial \text{Var}\left(y_{t(i)}\right)}{\partial \beta_{K,j}}=x_{t(i),j}\cdot \sum_{u=0}^{K-1}\left(p_{t(i),\leq u}\cdot \left(1-p_{t(i),\leq u}\right)\cdot \left(v_{u}-v_{u+1}\right)\right.\;\;\;\;\;\;\;\;\;\;\;\;\;\;\;\;\;\;\;\;\;\;\;\;\;\;\left.\;\;\;\;\;\;\cdot \left(v_{u}+v_{u+1}-2\cdot \mu_{t(i)}\right)\right),$$

while ***stdex***_*w*_ and ***stdex***_*K*,*j*_ are the *N* × 1 vectors with entries

$${\text{stdex}}_{t(i),w}=\frac{\partial \mu_{t(i)}}{\partial \alpha_{w}}/\sigma_{t(i)}+stde_{t(i)}\cdot W_{t(i),w}/2,$$

$$\frac{\partial \mu_{t(i)}}{\partial \alpha_{w}}=p_{t(i),\leq w}\cdot \left(1-p_{t(i),\leq w}\right)\cdot \left(v_{w}-v_{w+1}\right),$$

$${\text{stdex}}_{t(i),K,j}=\frac{\partial \mu_{t(i)}}{\partial \beta_{K,j}}/\sigma_{t(i)}+stde_{t(i)}\cdot W_{t(i),K,j}/2,$$

$$\frac{\partial \mu_{t(i)}}{\partial \beta_{K,j}}=x_{t(i),j}\cdot \sum_{u=0}^{K-1}\left(p_{t(i),\leq u}\cdot \left(1-p_{t(i),\leq u}\right)\cdot \left(v_{u}-v_{u-1}\right)\right),$$

for 0 ≤ *w* ≤ *K*, 1 ≤ *j* ≤ *J*, and t(i)∈T. ***H*** (***β)*** has entries

$$H_{w,w^{\prime }}(\beta )=-{\mathrm{stdexx}}_{w,w^{\prime }}^{\text{T}}\cdot R^{-1}(\rho )\cdot \mathrm{stde}-{\mathrm{stdex}}_{w}^{\text{T}}\cdot R^{-1}(\rho )\cdot {\mathrm{stdex}}_{w^{\prime }}\;\;\;\;\;\;\;-\sum_{i=1}^{N}W_{t(i),w,w^{\prime }}/2,$$

$$H_{w,K,j}(\beta )=-{\mathrm{stdexx}}_{w,K,j}^{\text{T}}\cdot R^{-1}(\rho )\cdot \mathrm{stde}-{\mathrm{stdex}}_{w}^{\text{T}}\cdot R^{-1}(\rho )\cdot {\mathrm{stdex}}_{K,j}\;\;\;\;\;\;\;-\sum_{i=1}^{N}W_{t(i),w,K,j}/2,$$

$$H_{K,j,w^{\prime }}(\beta )=-{\mathrm{stdexx}}_{K,j,w^{\prime }}^{\text{T}}\cdot R^{-1}(\rho )\cdot \mathrm{stde}-{\mathrm{stdex}}_{K,j}^{\text{T}}\cdot R^{-1}(\rho )\cdot {\mathrm{stdex}}_{w^{\prime }}\;\;\;\;\;\;\;-\sum_{i=1}^{N}W_{t(i),K,j,w^{\prime }}/2,$$

$$H_{K,j,j^{\prime }}(\beta )=-{\mathrm{stdexx}}_{K,j,j^{\prime }}^{\text{T}}\cdot R^{-1}(\rho )\cdot \mathrm{stde}-{\mathrm{stdex}}_{K,j}^{\text{T}}\cdot R^{-1}(\rho )\cdot {\mathrm{stdex}}_{K,j^{\prime }}\;\;\;\;\;\;\;-\sum_{i=1}^{N}W_{t(i),K,j,j^{\prime }}/2,$$

Where

$$W_{t(i),w,w^{\prime }}=\frac{\partial W_{t(i),w}}{\partial \alpha_{w^{\prime }}}=\frac{\frac{\partial^{2}\text{Var}\left(y_{t(i)}\right)}{\partial \alpha_{w}\partial \alpha_{w^{\prime }}}}{\text{Var}\left(y_{t(i)}\right)}-\frac{\frac{\partial \text{Var}\left(y_{t(i)}\right)}{\partial \alpha_{w^{\prime }}}\cdot \frac{\partial \text{Var}\left(y_{t(i)}\right)}{\partial \alpha_{w}}}{{\text{Var}}^{2}\left(y_{t(i)}\right)},$$

$$\frac{\partial^{2}\text{Var}\left(y_{t(i)}\right)}{\partial \alpha_{w}\partial \alpha_{w}}\;=p_{t(i),\leq w}\cdot \left(1-p_{t(i),\leq w}\right)\cdot \left(v_{w}-v_{w+1}\right)\cdot \left(\left(1-2\cdot p_{t(i),\leq w}\right)\cdot \left(v_{w}+v_{w+1}-2\cdot \mu_{t(i)}\right)\right.\;\left.\;\;\;\;-2\cdot p_{t(i),\leq w}\cdot \left(1-p_{t(i),\leq w}\right)\cdot \left(v_{w}-v_{w+1}\right)\right),w^{\prime }=w,$$

$$\frac{\partial^{2}\text{Var}\left(y_{t(i)}\right)}{\partial \alpha_{w^{\prime }}\partial \alpha_{w}}=-2\cdot p_{t(i),\leq w}\cdot \left(1-p_{t(i),\leq w}\right)\cdot p_{t(i),\leq w^{\prime }}\cdot \left(1-p_{t(i),\leq w^{\prime }}\right)\;\;\;\;\;\;\;\;\;\;\;\;\;\;\;\;\;\;\;\;\;\;\;\;\;\;\;\;\;\;\;\;\;\;\cdot \left(v_{w}-v_{w+1}\right)\cdot \left(v_{w^{\prime }}-v_{w^{\prime }+1}\right),w^{\prime }\neq w;$$

$$W_{t(i),w,K,j}=\frac{\partial W_{t(i),w}}{\partial \beta_{K,j}}=\frac{\frac{\partial^{2}\text{Var}\left(y_{t(i)}\right)}{\partial \beta_{K,j}\partial \alpha_{w}}}{\text{Var}\left(y_{t(i)}\right)}-\frac{\frac{\partial \text{Var}\left(y_{t(i)}\right)}{\partial \beta_{K,j}}\cdot \frac{\partial \text{Var}\left(y_{t(i)}\right)}{\partial \alpha_{w}}}{{\text{Var}}^{2}\left(y_{t(i)}\right)},$$

$$\frac{\partial^{2}Var\left(y_{t(i)}\right)}{\partial \beta_{K,j}\partial \alpha_{w}}\;=x_{t(i),j}\cdot p_{t(i),\leq w}\cdot \left(1-p_{t(i),\leq w}\right)\cdot \left(v_{w}-v_{w+1}\right)\cdot \left(\left(1-2\cdot p_{t(i),\leq w}\right)\right.\;\left.\;\;\;\;\cdot \left(v_{w}+v_{w+1}-2\cdot \mu_{t(i)}\right)-2\cdot \sum_{u=0}^{K-1}\left(p_{t(i),\leq u}\cdot \left(1-p_{t(i),\leq u}\right)\cdot \left(v_{u}-v_{u+1}\right)\right)\right);$$

$$W_{t(i),K,j,w^{\prime }}=\frac{\partial W_{t(i),K,j}}{\partial \alpha_{w^{\prime }}}=\frac{\frac{\partial^{2}\text{Var}\left(y_{t(i)}\right)}{\partial \alpha_{w^{\prime }}\beta_{K,j}}}{\text{Var}\left(y_{t(i)}\right)}-\frac{\frac{\partial \text{Var}\left(y_{t(i)}\right)}{\partial \alpha_{w^{\prime }}}\cdot \frac{\partial \text{Var}\left(y_{t(i)}\right)}{\partial \beta_{K,j}}}{{\text{Var}}^{2}\left(y_{t(i)}\right)},$$

$$\frac{\partial^{2}\text{Var}\left(y_{t(i)}\right)}{\partial \alpha_{w}\partial \beta_{K,j}}\;=x_{t(i),j}\cdot p_{t(i),\leq w^{\prime }}\cdot \left(1-p_{t(i),\leq w^{\prime }}\right)\cdot \left(v_{w^{\prime }}-v_{w^{\prime }+1}\right)\cdot \left(\left(1-2\cdot p_{t(i),\leq w^{\prime }}\right)\right.\;\;\;\;\;\left.\cdot \left(v_{w^{\prime }}+v_{w^{\prime }+1}-2\cdot \mu_{t(i)}\right)-2\cdot \sum_{u=0}^{K-1}\left(p_{t(i),\leq u}\cdot \left(1-p_{t(i),\leq u}\right)\cdot \left(v_{u}-v_{u+1}\right)\right)\right);$$

$$W_{t(i),K,j,j^{\prime }}=\frac{\partial W_{t(i),K,j}}{\partial \beta_{K,j^{\prime }}}=\frac{\frac{\partial^{2}\text{Var}\left(y_{t(i)}\right)}{\partial \beta_{K,j^{\prime }}\partial \beta_{K,j}}}{\text{Var}\left(y_{t(i)}\right)}-\frac{\frac{\partial \text{Var}\left(y_{t(i)}\right)}{\partial \beta_{K,j^{\prime }}}\cdot \frac{\partial \text{Var}\left(y_{t(i)}\right)}{\partial \beta_{K,j}}}{{\text{Var}}^{2}\left(y_{t(i)}\right)}$$

$$\frac{\partial^{2}\text{Var}\left(y_{t(i)}\right)}{\partial \beta_{K,j^{\prime }}\partial \beta_{K,j}}\;=x_{t(i),j}\cdot x_{t(i),j^{\prime }}\cdot \sum_{u=0}^{K-1}\left(p_{t(i),\leq u}\cdot \left(1-p_{t(i),\leq u}\right)\cdot \left(1-2\cdot p_{t(i),\leq u}\right)\right.\;\left.\;\;\;\cdot \left(v_{u}-v_{u+1}\right)\cdot \left(v_{u}+v_{u+1}-2\cdot \mu_{t(i)}\right)\right)\;\;\;-2\cdot x_{t(i),j}\cdot x_{t(i),j^{\prime }}\cdot {\left(\sum_{u=0}^{K-1}\left(p_{t(i),\leq u}\cdot \left(1-p_{t(i),\leq u}\right)\cdot \left(v_{u}-v_{u+1}\right)\right)\right)}^{2}$$

while ${\mathrm{stdexx}}_{w,w^{\prime }}$, ${\mathrm{stdexx}}_{w,K,j}$, ${\mathrm{stdexx}}_{K,j,w^{\prime }}$ and ${\mathrm{stdexx}}_{K,j,j^{\prime }}$ are the *N* × 1 vectors with respective entries

$${\text{stdexx}}_{t(i),w,w^{\prime }}=-\frac{\frac{\partial^{2}\mu_{t(i)}}{\partial \alpha_{w^{\prime }}\partial \alpha_{w}}}{\sigma_{t(i)}}+\frac{\partial \mu_{t(i)}}{\partial \alpha_{w}}\cdot \frac{W_{t(i),w^{\prime }}}{2\cdot \sigma_{t(i)}}+{\text{stdex}}_{t(i),w^{\prime }}\cdot \frac{W_{t(i),w}}{2}\;\;\;\;\;\;\;\;\;\;\;\;\;\;\;\;\;\;\;\;\;\;\;\;\;\;\;\;\;\;\;-stde_{t(i)}\cdot \frac{W_{t(i),w,w^{\prime }}}{2},$$

$$\frac{\partial^{2}\mu_{t(i)}}{\partial \alpha_{w^{\prime }}\partial \alpha_{w}}=p_{t(i),\leq w}\cdot \left(1-p_{t(i),\leq w}\right)\cdot \left(1-2\cdot p_{t(i),\leq w}\right)\cdot \left(v_{w}-v_{w+1}\right),w^{\prime }=w,$$

$$\frac{\partial^{2}\mu_{t(i)}}{\partial \alpha_{w^{\prime }}\partial \alpha_{w^{\prime }}}=0,w^{\prime }\neq w;$$

$${\text{stdexx}}_{t(i),w,K,j}=-\frac{\frac{\partial^{2}\mu_{t(i)}}{\partial \beta_{K,j}\partial \alpha_{w}}}{\sigma_{t(i)}}+\frac{\partial \mu_{t(i)}}{\partial \alpha_{w}}\cdot \frac{W_{t(i),K,j}}{2\cdot \sigma_{t(i)}}+{\text{stdex}}_{t(i),K,j}\cdot \frac{W_{t(i),w}}{2}\;\;\;\;\;\;\;\;\;\;\;\;\;\;\;\;\;\;\;\;\;\;\;\;\;\;\;\;\;\;\;\;\;-stde_{t(i)}\cdot \frac{W_{t(i),w,K,j}}{2},$$

$$\frac{\partial^{2}\mu_{t(i)}}{\partial \beta_{K,j}\partial \alpha_{w}}=x_{t(i),j}\cdot p_{t(i),\leq w}\cdot \left(1-p_{t(i),\leq w}\right)\cdot \left(1-2\cdot p_{t(i),\leq w}\right)\cdot \left(v_{w}-v_{w+1}\right);$$

$${\text{stdexx}}_{t(i),K,j,w^{\prime }}=-\frac{\frac{\partial^{2}\mu_{t(i)}}{\partial \alpha_{w^{\prime }}\beta_{K,j}}}{\sigma_{t(i)}}+\frac{\partial \mu_{t(i)}}{\partial \beta_{K,j}}\cdot \frac{W_{t(i),w^{\prime }}}{2\cdot \sigma_{t(i)}}+{\text{stdex}}_{t(i),w^{\prime }}\cdot \frac{W_{t(i),K,j}}{2}\;\;\;\;\;\;\;\;\;\;\;\;\;\;\;\;\;\;\;\;\;\;\;\;\;\;\;\;\;\;\;\;\;\;-stde_{t(i)}\cdot \frac{W_{t(i),K,j,w^{\prime }}}{2},$$

$$\frac{\partial^{2}\mu_{t(i)}}{\partial \alpha_{w^{\prime }}\partial \beta_{K,j}}=x_{t(i),j}\cdot p_{t(i),\leq w^{\prime }}\cdot \left(1-p_{t(i),\leq w^{\prime }}\right)\cdot \left(1-2\cdot p_{t(i),\leq w^{\prime }}\right)\cdot \left(v_{w^{\prime }}-v_{w^{\prime }+1}\right);$$

$${\text{stdexx}}_{t(i),K,j,j^{\prime }}=-\frac{\frac{\partial^{2}\mu_{t(i)}}{\partial \beta_{K,j^{\prime }}\partial \beta_{K,j}}}{\sigma_{sc}}+\frac{\partial \mu_{t(i)}}{\partial \beta_{K,j}}\cdot \frac{W_{t(i),K,j^{\prime }}}{2\cdot \sigma_{sc}}+{\text{stdex}}_{t(i),K,j^{\prime }}\cdot \frac{W_{t(i),K,j}}{2}\;\;\;\;\;\;\;\;\;\;\;\;\;\;\;\;\;\;\;\;\;\;\;\;\;\;\;\;\;\;\;\;\;\;-stde_{t(i)}\cdot \frac{W_{t(i),K,j,j^{\prime }}}{2},$$

$$\frac{\partial^{2}\mu_{t(i)}}{\partial \beta_{K,j^{\prime }}\partial \beta_{K,j}}=x_{t(i),j}\cdot x_{t(i),j^{\prime }}\cdot \sum_{u=0}^{K-1}\left(p_{t(i),\leq u}\cdot \left(1-p_{t(i),\leq u}\right)\right.\;\;\;\;\;\;\;\;\;\;\;\;\;\;\;\;\;\;\;\;\;\;\;\;\;\;\;\;\;\left.\cdot \left(1-2\cdot p_{t(i),\leq u}\right)\cdot \left(v_{u}-v_{u+1}\right)\right);$$

for $0\leq w,w^{\prime }<K$ and $1\leq j,j^{\prime }\leq J$. $H(\beta ,\beta^{\prime })$ has entries

$$H_{w,j^{\prime }}(\beta ,\beta^{\prime })=-{\mathrm{stdexx}}_{w,j^{\prime }}^{\prime \text{T}}\cdot R^{-1}(\rho )\cdot \mathrm{stde}-{\mathrm{stdex}}_{w}^{\text{T}}\cdot R^{-1}(\rho )\cdot {\mathrm{stdex}}_{j^{\prime }},$$

$$H_{K,j,j^{\prime }}(\beta ,\beta^{\prime })=-{\mathrm{stdexx}}_{K,j,j^{\prime }}^{\prime \text{T}}\cdot R^{-1}(\rho )\cdot \mathrm{stde}-{\mathrm{stdex}}_{K,j}^{\text{T}}\cdot R^{-1}(\rho )\cdot {\mathrm{stdex}}_{j^{\prime }},$$

where ${\mathrm{stdexx}}_{w,j^{\prime }}^{\prime }$ and ${\mathrm{stdexx}}_{k,j,j^{\prime }}^{\prime }$ are the *N* × 1 vectors with respective entries

$${\mathrm{stdexx}}_{t(i),w,j^{\prime }}^{\prime }=\mathrm{stde}x_{t(i),w}\cdot x_{t(i),j^{\prime }}^{\prime }/2,$$

$${\mathrm{stdexx}}_{t(i),K,j,j^{\prime }}^{\prime }=\mathrm{stde}x_{t(i),K,j}\cdot x_{t(i),j^{\prime }}^{\prime }/2,$$

for 0≤w<K, 1≤j≤J, $1\leq j^{\prime }\leq J^{\prime }$, and t(i)∈T.***H*(*β*,** *ρ***)** has entries

$$H_{w}(\beta ,\rho )={\mathrm{stdex}}_{w}^{\text{T}}\cdot \frac{\partial R^{-1}(\rho )}{\partial \rho }\cdot \mathrm{stde},$$

$$H_{K,j}(\beta ,\rho )={\mathrm{stdex}}_{K,j}^{\text{T}}\cdot \frac{\partial R^{-1}(\rho )}{\partial \rho }\cdot \mathrm{stde},$$

for 0 ≤ *w* ≤ *K* and 1 ≤ *j* ≤ *J*.

### 2.3. Censored Poisson Probabilities

The censored Poisson probabilities

$$p_{t(i),u}=\text{P}\left(y_{t(i)}=v_{u}\right)$$

are modeled as follows

$$p_{t(i),u}=\exp\left(-\lambda_{t(i)}\right)\cdot \frac{\lambda_{t(i)}^{u}}{u!},0\leq u<K$$

$$p_{t(i),K}=1-\sum_{u=0}^{K}p_{t(i),u}$$

$$\log\lambda_{t(i)}=x_{t(i)}^{\text{T}}\cdot \beta ,$$

using the natural log link function for modeling $\lambda_{t(i)}$, for t(i)∈T. There are *J* coefficient parameters for modeling the probabilities. Setting $x_{t(i),j}=1$ for t(i)∈T generates an intercept parameter.

In the special case when *v*_*u*_ are consecutive integers, that is, *v*_*u*_ = *c* + *u* for an integer *c* ≥ 0 and 0 ≤ *u* < *K*, truncated Poisson probabilities [19] could be used instead with

$$p_{t(i),u}=\exp\left(-\lambda_{t(i)}\right)\cdot \frac{\lambda_{t(i)}^{c+u}}{(c+u)!\cdot S},0\leq u\leq K,$$

where the normalizing constant *S* satisfies

$$S=\sum_{u=0}^{K}\frac{\lambda_{t(i)}^{c+u}}{(c+u)!}.$$

These are not considered any further.

The first partial derivatives $\frac{\partial \lambda_{t(i)}}{\partial \beta_{j}}$ and $\frac{\partial p_{t(i),u}}{\partial \beta_{j}}$ satisfy

$$\frac{\partial \lambda_{t(i)}}{\partial \beta_{j}}=x_{t(i),j}\cdot \lambda_{t(i)},$$

$$\frac{\partial p_{t(i),u}}{\partial \beta_{j}}=x_{t(i),j}\cdot p_{t(i),u}\cdot \left(u-\lambda_{t(i)}\right),0\leq u<K,$$

$$\frac{\partial p_{t(i),K}}{\partial \beta_{j}}=-\sum_{u=0}^{K-1}\frac{\partial p_{t(i),u}}{\partial \beta_{j}},$$

for 1 ≤ *j* ≤ *r* and t(i)∈T. The associated second partial derivatives satisfy

$$\frac{\partial^{2}\lambda_{t(i)}}{\partial \beta_{j^{\prime }}\partial \beta_{j}}=x_{t(i),j}\cdot x_{t(i),j^{\prime }}\cdot \lambda_{t(i)},$$

$$\frac{\partial^{2}p_{t(i),u}}{\partial \beta_{j^{\prime }}\partial \beta_{j}}=x_{t(i),j}\cdot x_{t(i),j^{\prime }}\cdot p_{t(i),u}\cdot \left({\left(u-\lambda_{t(i)}\right)}^{2}-\lambda_{t(i)}\right),0\leq u<K,$$

$$\frac{\partial^{2}p_{t(i),K}}{\partial \beta_{j^{\prime }}\partial \beta_{j}}=-\sum_{u=0}^{K-1}\frac{\partial^{2}p_{t(i),u}}{\partial \beta_{j^{\prime }}\partial \beta_{j}},$$

for $1\leq j,j^{\prime }\leq r$ and t(i)∈T

The derivative vector ***H*** (***β)*** has *J* entries *g*_*j*_ (***β)*** for 1 ≤ *j* ≤ *J* satisfying

$$g_{j}(\beta )={\mathrm{stdex}}_{j}^{\text{T}}\cdot R^{-1}(\rho )\cdot \mathrm{stde}-\sum_{i=1}^{N}W_{t(i),j}/2$$

where

$$W_{t(i),j}=\frac{\frac{\partial \text{Var}\left(y_{t(i)}\right)}{\partial \beta_{j}}}{\text{Var}\left(y_{t(i)}\right)},$$

$$\frac{\partial \text{Var}\left(y_{t(i)}\right)}{\partial \beta_{j}}=\sum_{u=0}^{K-1}\left(\left(v_{u}-v_{K}\right)\cdot \left(v_{u}+v_{K}-2\cdot \mu_{t(i)}\right)\cdot \frac{\partial p_{t(i),u}}{\partial \beta_{j}}\right),$$

and ***stdex***_*j*_ is the *N* × 1 vector with entries

$$\mathrm{stde}x_{t(i),j}=\frac{\partial \mu_{t(i)}}{\partial \beta_{j}}/\sigma_{t(i)}+stde_{t(i)}\cdot W_{t(i),j}/2,$$

$$\frac{\partial \mu_{t(i)}}{\partial \beta_{j}}=\sum_{u=0}^{K-1}\left(v_{u}-v_{K}\right)\cdot \frac{\partial p_{t(i),u}}{\partial \beta_{j}},$$

for 1 ≤ *j* ≤ *J* and t(i)∈T.

***H*** (***β)*** has entries

$$H_{j,j^{\prime }}(\beta )-{\mathrm{stdexx}}_{j,j^{\prime }}^{\text{T}}\cdot R^{-1}(\rho )\cdot \mathrm{stde}-{\mathrm{stdex}}_{j}^{\text{T}}\cdot R^{-1}(\rho )\cdot \mathrm{stde}x_{j^{\prime }}-\sum_{i=1}^{N}W_{t(i),j,j^{\prime }}/2$$

where

$$W_{t(i),j,j^{\prime }}=\frac{\partial W_{t(i),j}}{\partial \beta_{j^{\prime }}}=\frac{\frac{\partial^{2}\text{Var}\left(y_{t(i)}\right)}{\partial \beta_{j^{\prime }}\partial \beta_{j}}}{\text{Var}\left(y_{t(i)}\right)}-\frac{\frac{\partial \text{Var}\left(y_{t(i)}\right)}{\partial \beta_{j^{\prime }}}\cdot \frac{\partial \text{Var}\left(y_{t(i)}\right)}{\partial \beta_{j}}}{{\text{Var}}^{2}\left(y_{t(i)}\right)},$$

$$\frac{\partial^{2}\text{Var}\left(y_{t(i)}\right)}{\partial \beta_{j}\partial \beta_{j^{\prime }}}=\sum_{u=0}^{K-1}\left(\left(v_{u}-v_{K}\right)\cdot \left(v_{u}+v_{K}-2\cdot \mu_{t(i)}\right)\cdot \frac{\partial^{2}p_{t(i),u}}{\partial \beta_{j^{\prime }}\partial \beta_{j}}\right.\;\left.\;\;\;\;\;\;\;\;\;\;\;\;\;\;\;\;\;\;\;\;\;\;\;\;\;\;\;\;\;\;\;\;\;-2\cdot \frac{\partial \mu_{t(i)}}{\partial \beta_{j^{\prime }}}\cdot \frac{\partial p_{t(i),u}}{\partial \beta_{j}}\right),$$

While ${\mathrm{stdexx}}_{j,j^{\prime }}$ is the *N* × 1 vector with entries

$${\text{stdexx}}_{t(i),j,j^{\prime }}=-\frac{\frac{\partial^{2}\mu_{t(i)}}{\partial \beta_{j^{\prime }}\partial \beta_{j}}}{\sigma_{t(i)}}+\frac{\partial \mu_{t(i)}}{\partial \beta_{j}}\cdot \frac{W_{t(i),j^{\prime }}}{2\cdot \sigma_{t(i)}}+\mathrm{stde}x_{t(i),j^{\prime }}\cdot \frac{W_{t(i),j}}{2}-stde_{t(i)}\cdot \frac{W_{t(i),j,j^{\prime }}}{2},$$

$$\frac{\partial^{2}\mu_{t(i)}}{\partial \beta_{j^{\prime }}\partial \beta_{j}}=\sum_{u=0}^{K-1}\left(\left(v_{u}-v_{K}\right)\cdot \frac{\partial^{2}p_{t(i),u}}{\partial \beta_{j^{\prime }}\partial \beta_{j}}\right),$$

for $1\leq j,j^{\prime }\leq J$ and t(i)∈T. $H(\beta ,\beta^{\prime })$ has entries

$$H_{j,j^{\prime }}(\beta ,\beta^{\prime })=-{\mathrm{stdexx}}_{j,j^{\prime }}^{\prime \text{T}}\cdot R^{-1}(\rho )\cdot \mathrm{stde}-{\mathrm{stdex}}_{j}^{\text{T}}\cdot R^{-1}(\rho )\cdot {\mathrm{stdex}}_{j^{\prime }}^{\prime }$$

where ${\mathrm{stdexx}}_{j,j^{\prime }}^{\prime }$ is the *N* × 1 vector with entries

$${\mathrm{stdexx}}_{t(i),j,j^{\prime }}^{\prime }=\mathrm{stde}x_{t(i),j}\cdot x_{t(i),j^{\prime }}^{\prime }/2$$

for 1≤j≤J, $1\leq j^{\prime }\leq J^{\prime }$, and t(i)∈T. ***H*(*β*,** *ρ***)** has entries

$$H_{j}(\beta ,\rho )={\mathrm{stdex}}_{j}^{\text{T}}\cdot \frac{\partial R^{-1}(\rho )}{\partial \rho }\cdot \mathrm{stde}\;$$

for 1 ≤ *j* ≤ *J*.

### 2.4. Likelihood-Like Cross-Validation

In *k*-fold cross-validation [20], observations are partitioned into *k* disjoint subsets called folds. Parameter estimates computed using the data from the other folds are used to predict fold observations. In *k*-fold likelihood-like cross-validation (LCV), these deleted fold predictions are scored using the associated likelihood-like function *L*. The times t(i)∈T are randomly partitioned into *k* disjoint folds *T* (*f*) for 1 ≤ *f* ≤ *k*. The same initial seed is used for randomization with all models under consideration to generate compatible LCV scores. Denote the deleted estimate of ***θ*** using the data with times in the complement *T* \ *T* (*f*) of the fold *T* (*f*) as ***θ*** (*T* \ *T* (*f*)). For 1 ≤ *f* ≤ *k*, denote the union of all folds $T(f^{\prime })$ for $1\leq f^{\prime }\leq f$ as *T* ^+^ (*f*). *T* ^+^ (0) is the empty fold satisfying *L* (*T* ^+^ (0)) = 1. The LCV score is

$$\text{LCV}=\prod_{f=1}^{k}{\text{LCV}}_{f}^{1/N}$$

where LCV_*f*_ is the conditional likelihood-like term for the data in fold *T* (*f*) conditioned on the data in the union *T* ^+^ (*f* – 1) of the prior folds using the deleted estimate ***θ*** (*T* \ *T* (*f*)) of the parameter vector ***θ***. Formally,

$$LCV_{f}=L\left(T(f)|T^{+}(f-1);\theta (T\setminus T(f))\right)=\frac{L\left(T^{+}(f);\theta (T\setminus T(f))\right)}{L\left(T^{+}(f-1);\theta (T\setminus T(f))\right)}.$$

Larger LCV scores indicate better models.

### 2.5. Adaptive ELMM

Knafl and Ding [12] formulate adaptive regression methods for searching through alternative models for means and dispersions in a variety of contexts. These methods use adaptive fractional polynomial models [21]. A short overview is provided here (for details, see Chapter 20, [12]). Adaptive regression methods generalize to ELMM modeling of discrete outcomes and are used in the example analyses of individual-patient pain ratings (Section 3). Model selection is a two-phase heuristic process. First, the model is expanded (or grown) by adding power transforms of predictors for means and dispersions. Then, the model is contracted (or pruned) to a parsimonious set of power transforms by removing transforms from the current model one at a time and adjusting the powers of the remaining transforms. LCV scores are used to evaluate and compare alternative models. Tolerance parameters control the adaptive modeling process. These tolerance parameters indicate how much of a reduction in the LCV score can be tolerated at given stages of the process. Predictors having arbitrary values raised to arbitrary powers can generate floating point overflow problems. To counter this problem, power transformed predictor values are upper bounded to be no larger than 10^12^.

The adaptive modeling process can optionally generate geometric combinations, that is, products of power transforms of multiple predictors generalizing standard interactions, possibly with the geometric combinations also power transformed, for example, ${\left(x_{1}^{p}\cdot x_{2}^{p^{\prime }}\right)}^{p^{\prime \prime }}$. This provides for an assessment of nonlinear moderation, generalizing the standard linear form of moderation [22].

A wide variety of example analyses are provided in [12] demonstrating the usefulness of adaptive regression methods. However, adaptive modeling of discrete outcomes has not been previously addressed.

A SAS® (SAS Institute, Inc., Cary, NC) macro has been developed for conducting adaptive analyses. This macro as well as data and code used to generate the results of the example analyses along with SAS output for those analyses are available from the first author.

### 2.6. On-Going Study of Cancer Pain

The data analyzed in the example analyses have been collected as part of an on-going study of daily pain and opioid usage for cancer patients. This study is collecting a variety of measures including intensive longitudinal individual-patient data using Ecological Momentary Assessment (EMA) [23] as implemented in the mEMA app [24]. Each patient is providing data on numbers of pain flares, that is, sudden increases in pain, and of opioids taken on each day. Methods for analyzing such individual-patient longitudinal count outcomes using Poisson regression modeling are addressed in Knafl and Meghani [14]. Each patient is also providing data on ratings of worst pain and least pain on a scale of 0 – 10 for each day (as also used in the Brief Pain Inventory [4]). Methods for analyzing such individual-patient longitudinal pain rating data using discrete regression modeling are addressed above. The pain ratings for Cancer Patient 1 plotted in Figure 1 are daily worst pain ratings and are used in the analyses of Section 3. This on-going study received Institutional Review Board approval. All participants provided written informed consent.

## 3. Results of Example Analyses

Table 1 contains results for adaptive models for probabilities and dispersions of pain ratings for Cancer Patient 1 over time (as plotted in Figure 1) computed using ELMM and the spatial AR1 correlation structure. LCV scores are based on *k* = 5 folds with fold sizes ranging from 13 to 20 measurements. Multinomial and ordinal probabilities are based on a single power transform of time while censored Poisson probabilities are based on two power transforms of time. All three probability types have zero intercept terms, meaning that eight intercepts are zero for the multinomial probabilities, the first intercept is zero for the ordinal probabilities, and the one intercept is zero for the censored Poisson probabilities. All three models have dispersions based on a single transform of time with zero intercept terms. The multinomial model has nine parameters (eight slopes for the probability time transform and one slope for the dispersion transform), the ordinal model also has nine parameters (seven intercept parameters for the probabilities, one slope for the probability time transform, and one slope for the dispersion transform), the censored Poisson model has three parameters (one slope for each of two probability time transforms and one slope for the dispersion transform).

The multinomial model has the best (largest) LCV score 0.23083 while the ordinal model has the worst (smallest) LCV score 0.22635. The censored Poisson model has the intermediate LCV score 0.22935, but it is not much smaller than the LCV score for the multinomial model and is based on one-third the number of parameters. Furthermore, the censored model requires only 5.2 minutes of clock time compared to 25.9 or about 5.0 times more for the ordinal model and 42.7 minutes or about 8.2 times more for the multinomial model. Consequently, censored Poisson probabilities are preferable to the other two approaches for modeling the pain ratings of Cancer Patient 1 because they generate a competitive LCV score, are more parsimonious, and require less time to compute. For this reason, only censored Poisson probabilities are considered in subsequent analyses of the pain ratings of Cancer Patient 1.

### 3.1. Assessment of the Number of Folds

It is possible that a larger number *k* of folds is more appropriate to use in analyzing the pain ratings of Cancer Patient 1. However, adaptive models for censored Poisson probabilities and dispersions using 10 and 15 folds have smaller LCV scores 0.22706 and 0.22378, respectively. Consequently, only *k* = 5 folds are used to compute LCV scores in subsequent analyses.

### 3.2. Independent versus Autoregressive Correlations

The adaptive model for censored Poisson probabilities and dispersions assuming independent correlations has LCV score 0.22349, smaller than the LCV score for the associated model of Table 1, indicating that the spatial AR1 correlation structure is the more appropriate choice. Consequently, only spatial AR1 correlations are considered in subsequent analyses of the pain ratings of Cancer Patient 1.

### 3.3. Assessment of Constant Dispersions

The adaptive model for censored Poisson probabilities using spatial AR1 correlations and assuming constant dispersions has LCV score 0.19309 smaller than the LCV score for the associated model of Table 1. Thus, dispersions for the pain ratings of Cancer Patient 1 are reasonably considered to be non-constant.

### 3.4. Assessing Linearity in Time

Using censored Poisson probabilities based on untransformed time, that is, linear in time, with an intercept, the adaptive model in time for the dispersions using spatial AR1 correlations has LCV score 0.20959 smaller than the LCV score for the associated model of Table 1. Thus, the censored Poisson probabilities for the pain ratings of Cancer Patient 1 are reasonably treated as nonlinear in time.

### 3.5. Adaptive Model in Time

**Figure 2.**
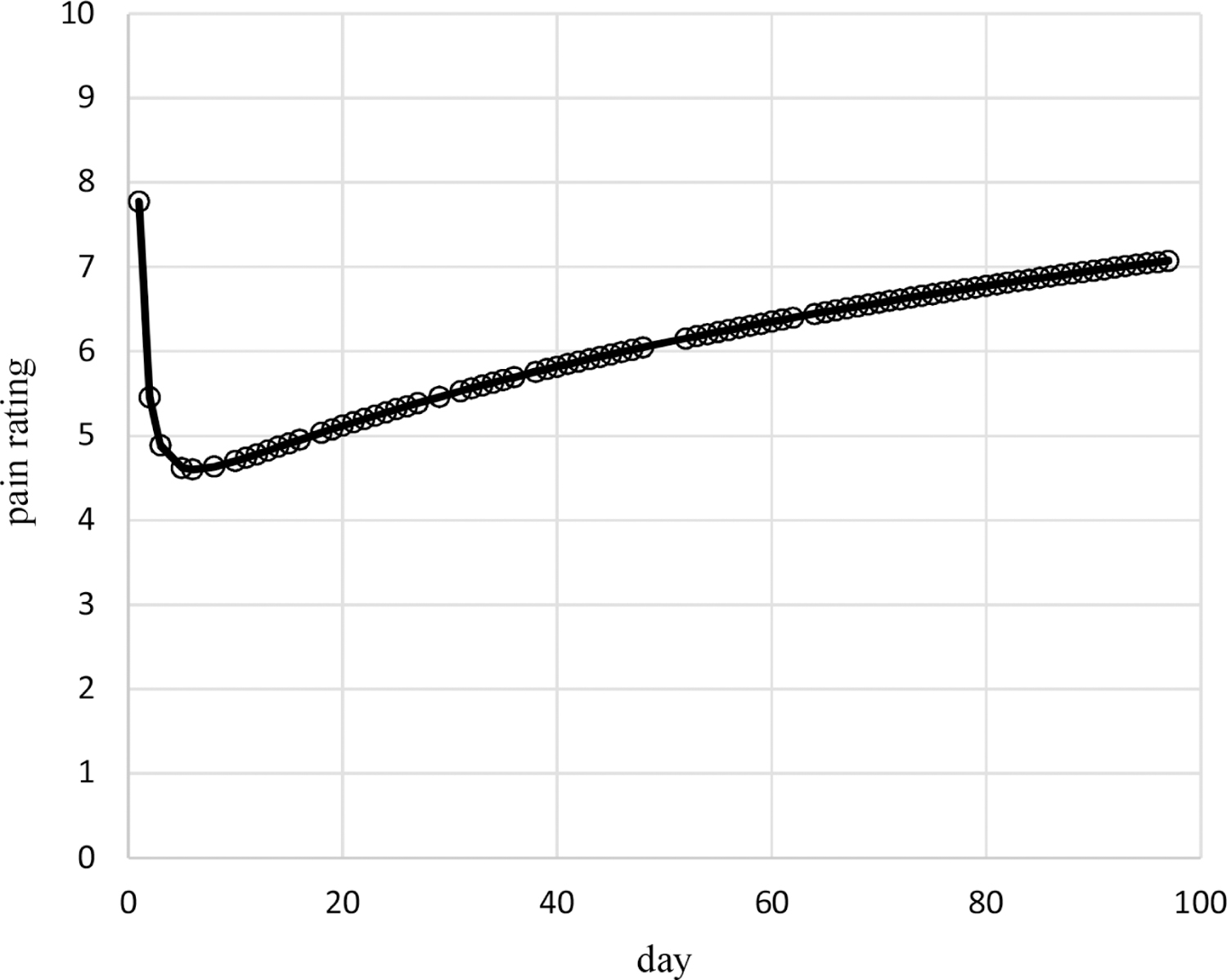
Estimated means of pain ratings over time for Cancer Patient 1.

**Figure 3.**
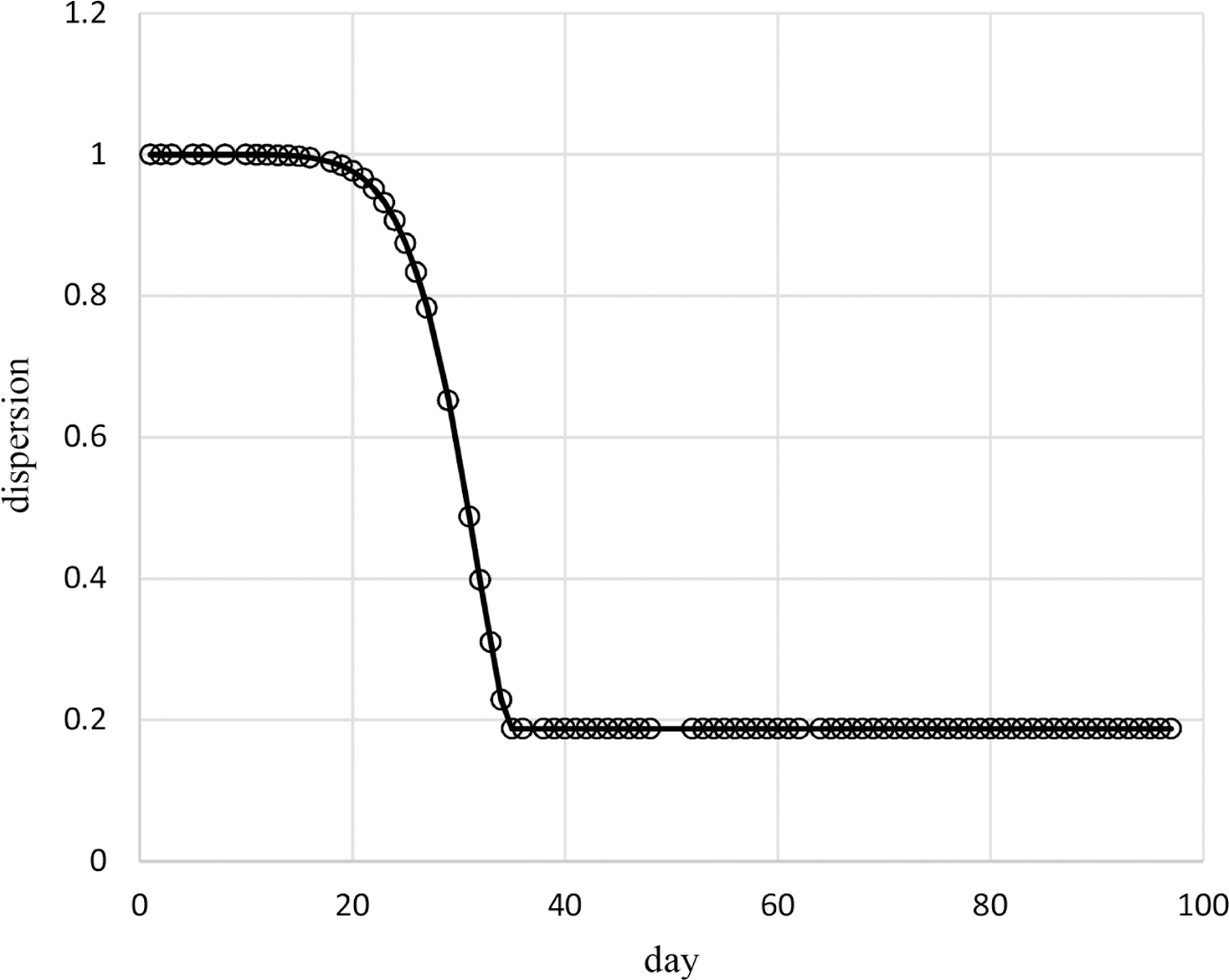
Estimated dispersions of pain ratings over time for Cancer Patient 1.

Results of the above analyses indicate that the censored Poisson model of Table 1 provides an appropriate assessment of the dependence on time of the probabilities and dispersions of the pain ratings of Cancer Patient 1. The probabilities for this model are based on time^0.2^ and time^−1^ without an intercept while the dispersions are based on time^7.8^ without an intercept. The estimated autocorrelation is 0.39 so that correlations decrease quickly with increased days apart, for example, the correlation is less than 0.01 for outcomes 5 or more days apart. Figure 2 contains the plot of estimated mean pain ratings over time, which decrease from 7.8 at day 1 quickly to 4.6 by day 6 and then increase to 7.1 by day 97. Figure 3 contains the plot of estimated dispersions for pain ratings over time, which decrease from 1 over days 1 – 15 to 0.19 at day 35, and remain constant after that (due to upper bounding the dispersion transform).

**Figure 4.**
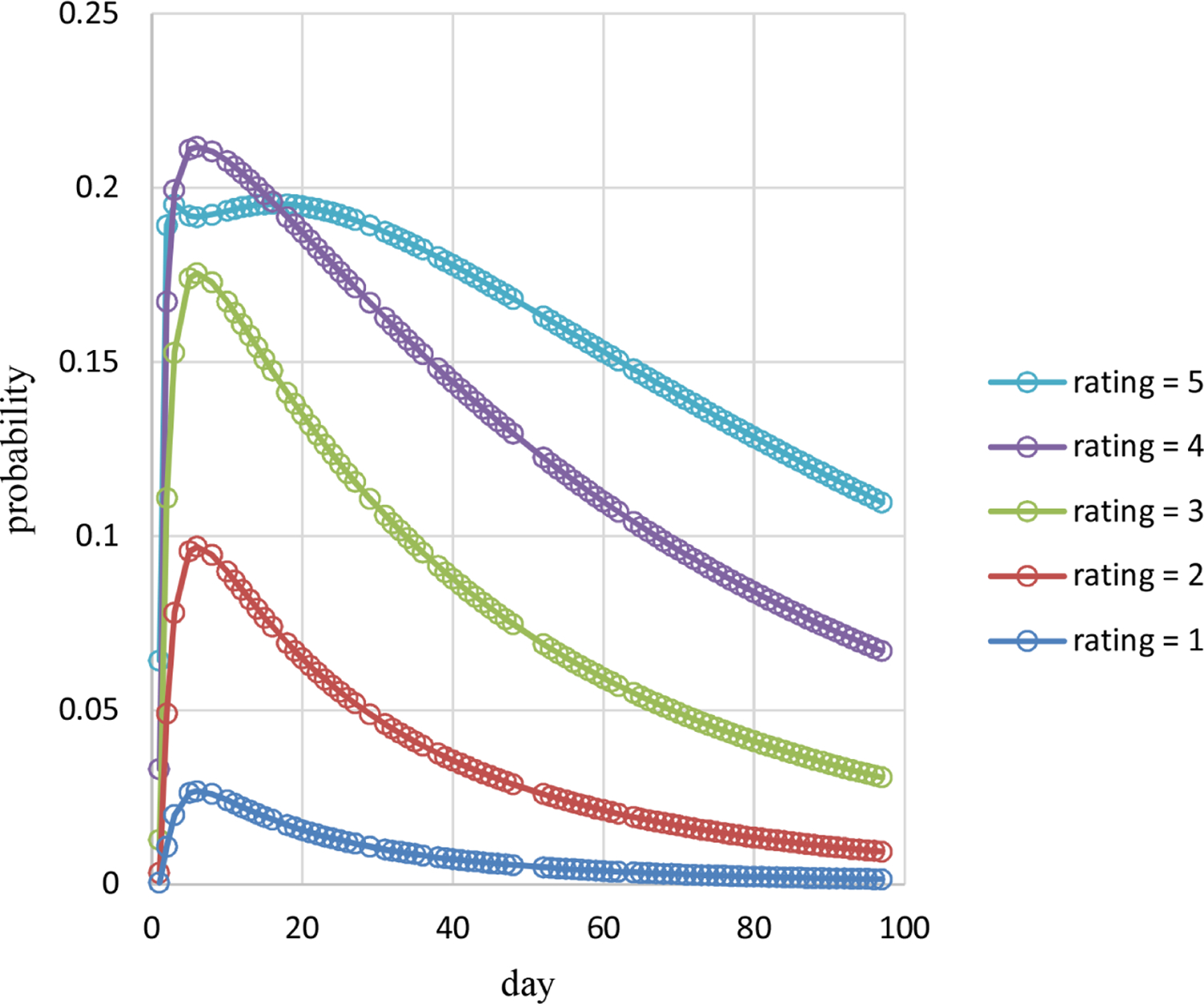
Estimated probabilities of pain ratings 1 – 5 over time for Cancer Patient 1.

**Figure 5.**
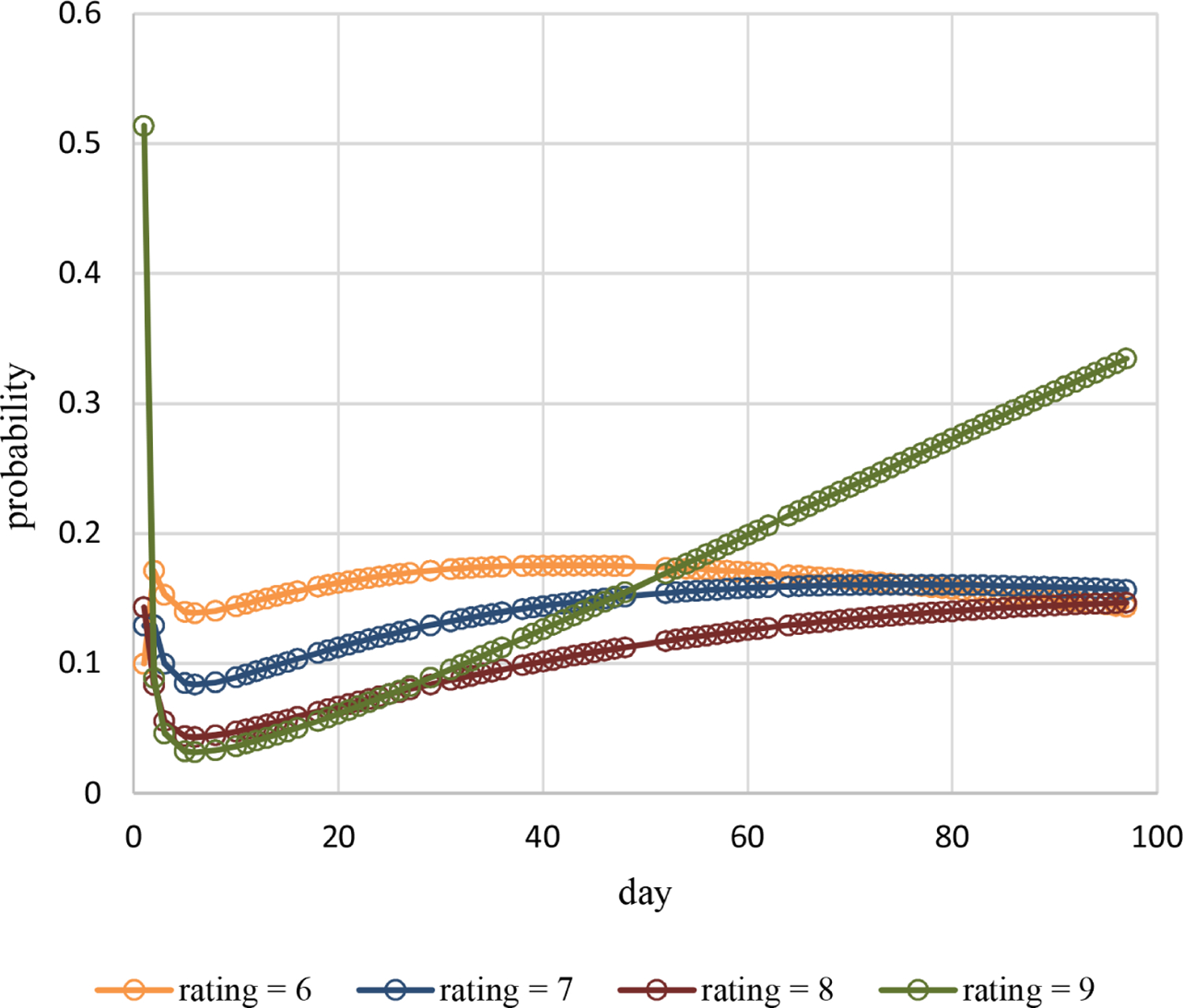
Estimated probabilities of pain ratings 6 – 9 over time for Cancer Patient 1.

**Figure 6.**
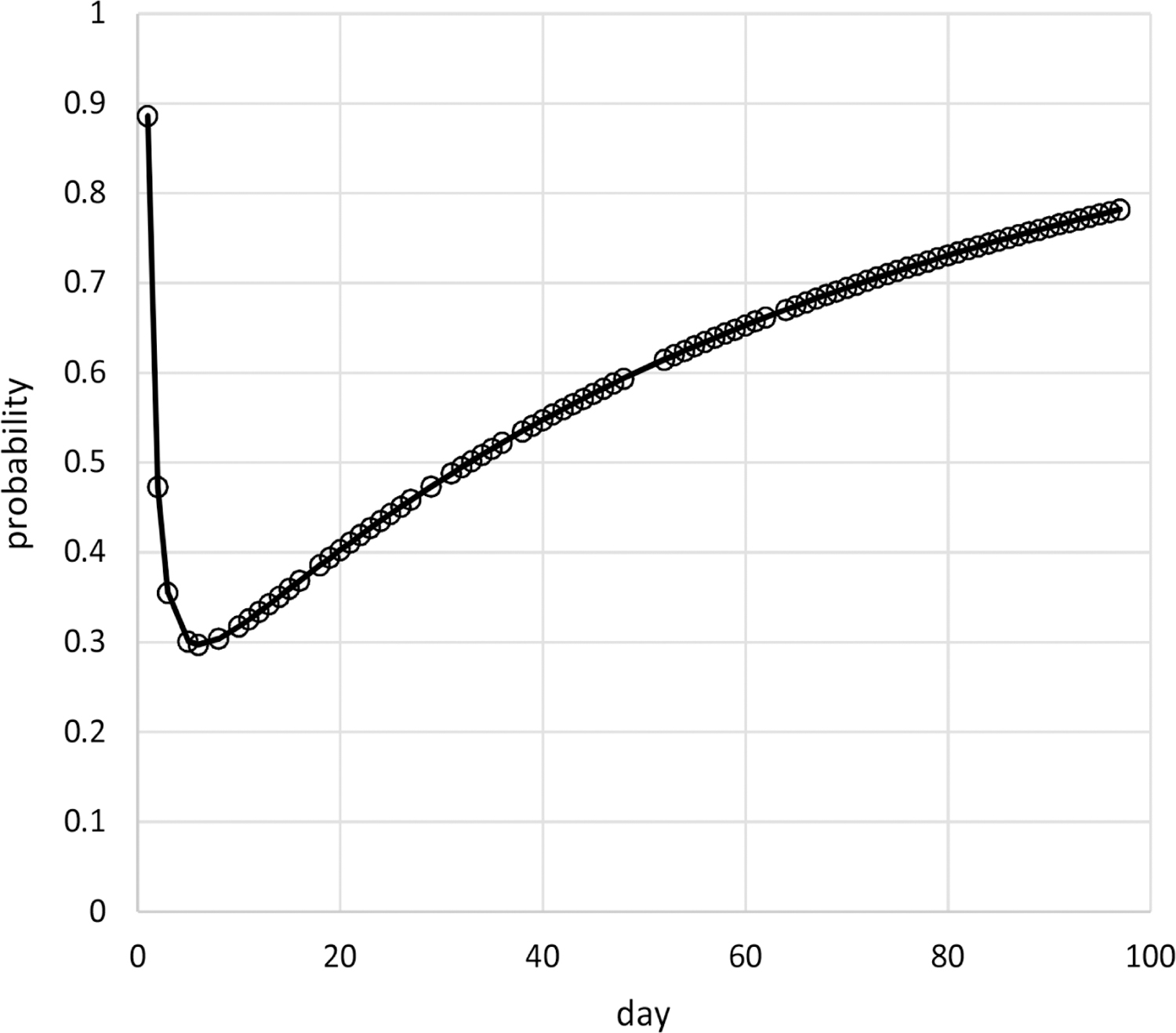
Estimated probabilities of a high pain rating of 6 or more over time for Cancer Patient 1.

Estimated probabilities over time for pain ratings 1 – 5 are plotted in Figure 4 and for pain ratings 6 – 9 in Figure 5. Estimated probabilities for pain ratings 1 – 5 increase quickly early on and decrease after that. Estimated probabilities for pain ratings 6 – 9 decrease quickly early on and increase after that with some small decreases late in time for pain ratings 6 – 7. Estimated probabilities are all smaller than 0.25 for pain ratings 1 – 8. The estimated probability of the highest observed pain rating of 9 is 0.51 on day 1, decreases quickly to 0.03 by day 6, and then increases to 0.33 by day 97. Estimated probabilities over time for a high pain rating of 6 or more are plotted in Figure 6. The estimated probability of a high pain rating of 6 or more starts at 0.89 on day 1, decreases to 0.30 by day 6, and then increases to 0.78 by day 97.

### 3.6. Adaptive Additive Model in Time and the Number of Pain Flares

Numbers of pain flares for Cancer Patient 1 vary from 0–4 with none missing for the *N* = 86 time points. By default, the adaptive modeling process generates additive models in multiple predictors. When applied to the pain ratings as a function of the number *x* of pain flares as well as of time, the generated additive model has probabilities based on a zero intercept, *x*^0.8^, and time^0.21^ ; dispersions based on a zero intercept and time^1.7^ ; estimated autocorrelation 0.37; and LCV score 0.28173. Since this LCV score is larger than the LCV score 0.22935 for the model based on only time, the number of pain flares is reasonably considered to have an additive effect on the censored Poisson probabilities, but not on the dispersions.

**Figure 7.**
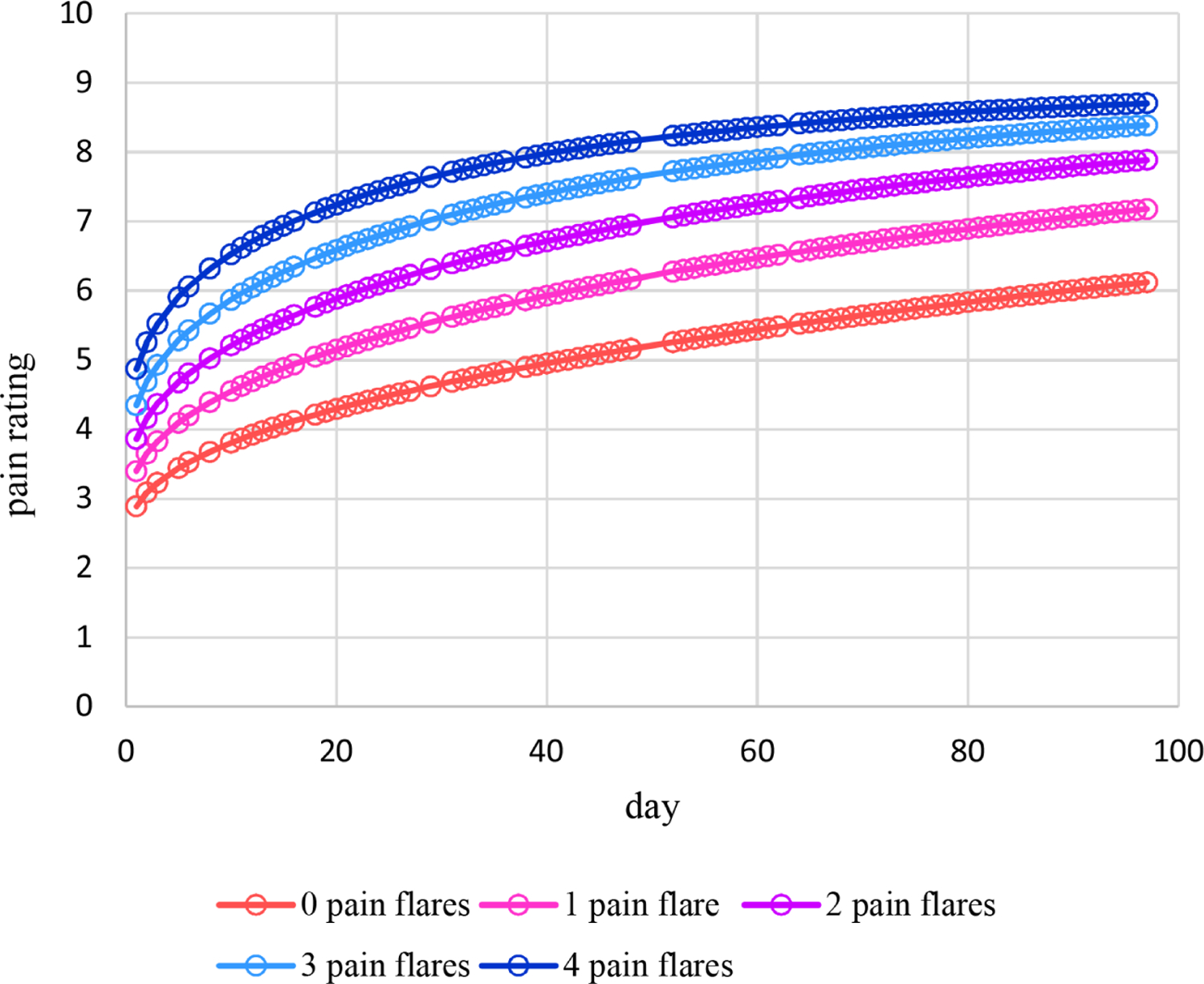
Estimated means of pain ratings over time and changing additively with the number of pain flares for Cancer Patient 1.

**Figure 8.**
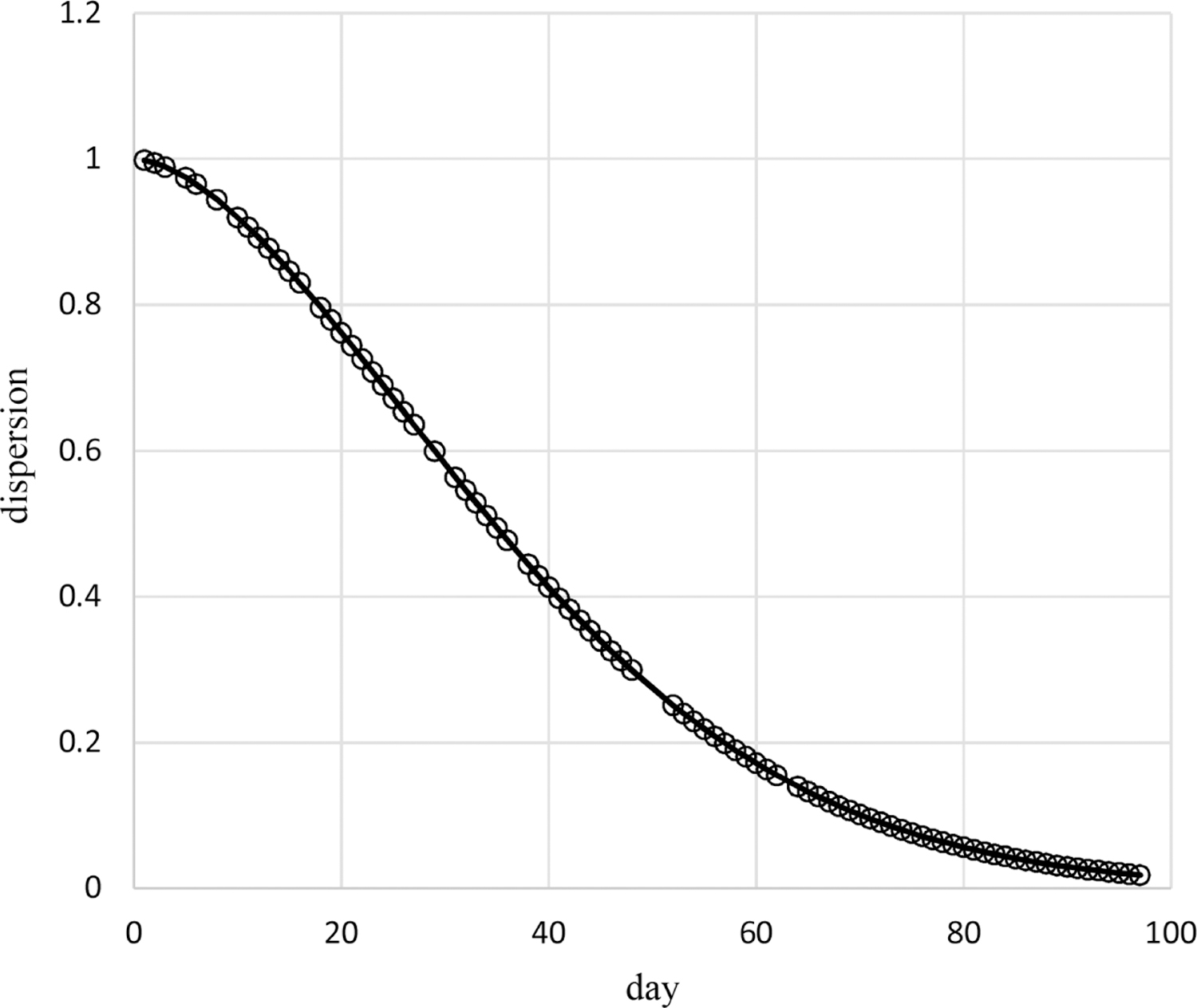
Estimated dispersions for pain ratings over time under the additive model for Cancer Patient 1.

Estimated means under this additive model are plotted in Figure 7, which increase nonlinearly over time at higher levels for higher numbers of pain flares. Figure 8 displays the plot of estimated dispersions for the additive model, which decrease nonlinearly from 1 at day 1 to 0.02 by day 97. Plots for estimated probabilities are not provided because that requires two plots similar to Figure 4 and Figure 5 for each of the five observed numbers of pain flares.

### 3.7. Adaptive Moderation of the Effect to Time by the Number of Pain Flares

Optionally, the adaptive modeling process can generate moderation models allowing for additive effects of multiple predictors together with geometric combinations based on those predictors. When applied to the pain ratings as a function of the number *x* of pain flares as well as of time, the generated moderation model has probabilities based on a zero intercept, *x*^2.7^, time^0.22^, and the four geometric combinations (time^4^ · *x*^−0.3^)^1.5^, (*x*^−5^ · time^1,.1^)^0.7^, (*x*^1.4^ · time^1,.1^)^1.6^, and (*x*^6^ · time^0.7^)^1.2^ ; dispersions based on a zero intercept, time^2^, and the one geometric combination (*x*^−2.5^ · time)^0.5^ ; estimated autocorrelation 0.32; and the LCV score is 0.29730. Since this LCV score is larger than the LCV score 0.28173 for the additive model, the number of pain flares is reasonably considered to moderate the effect of time on the censored Poisson probabilities as well as on the dispersions.

**Figure 9.**
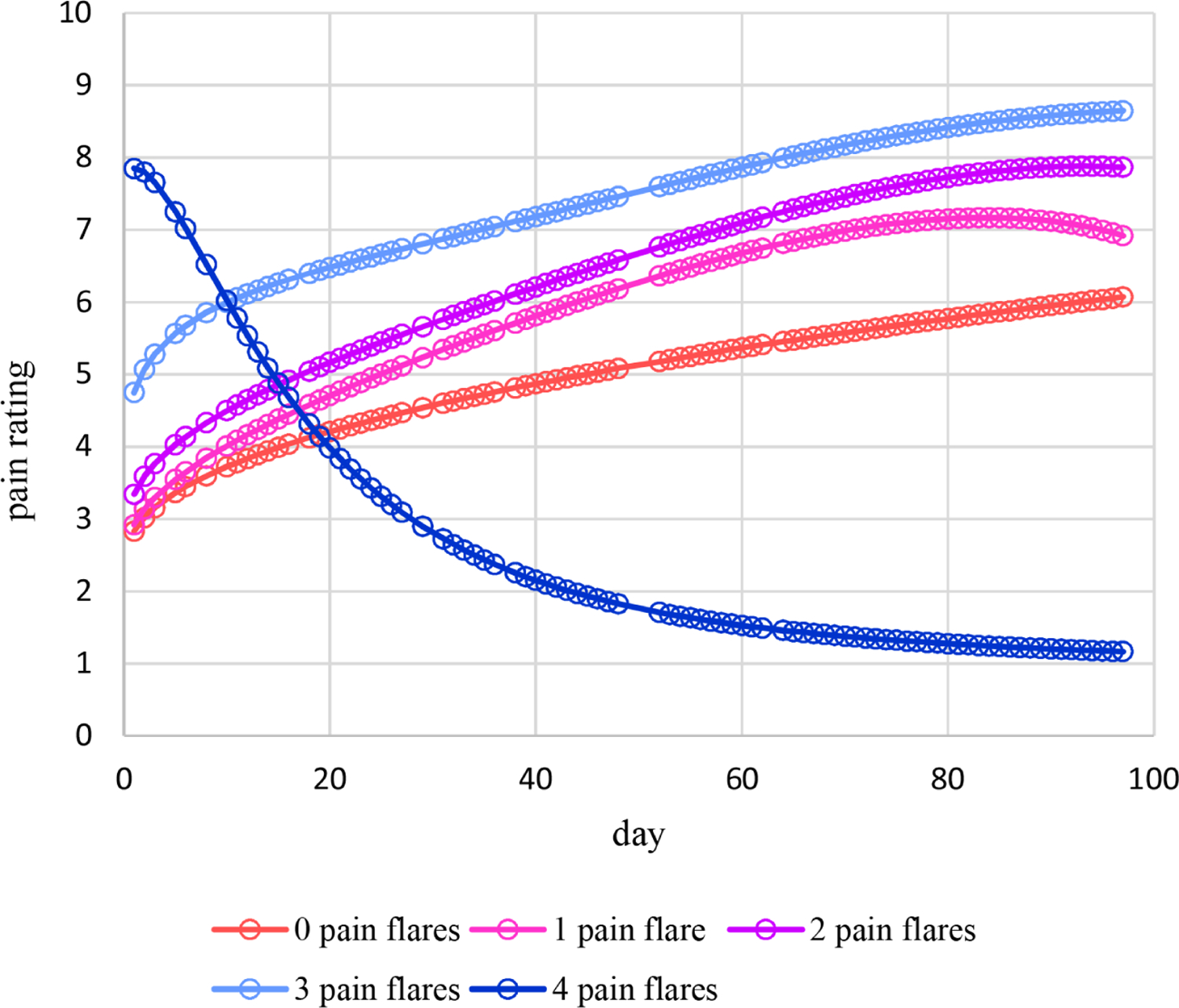
Estimated means of pain ratings over time moderated by the number of pain flares for Cancer Patient 1.

**Figure 10.**
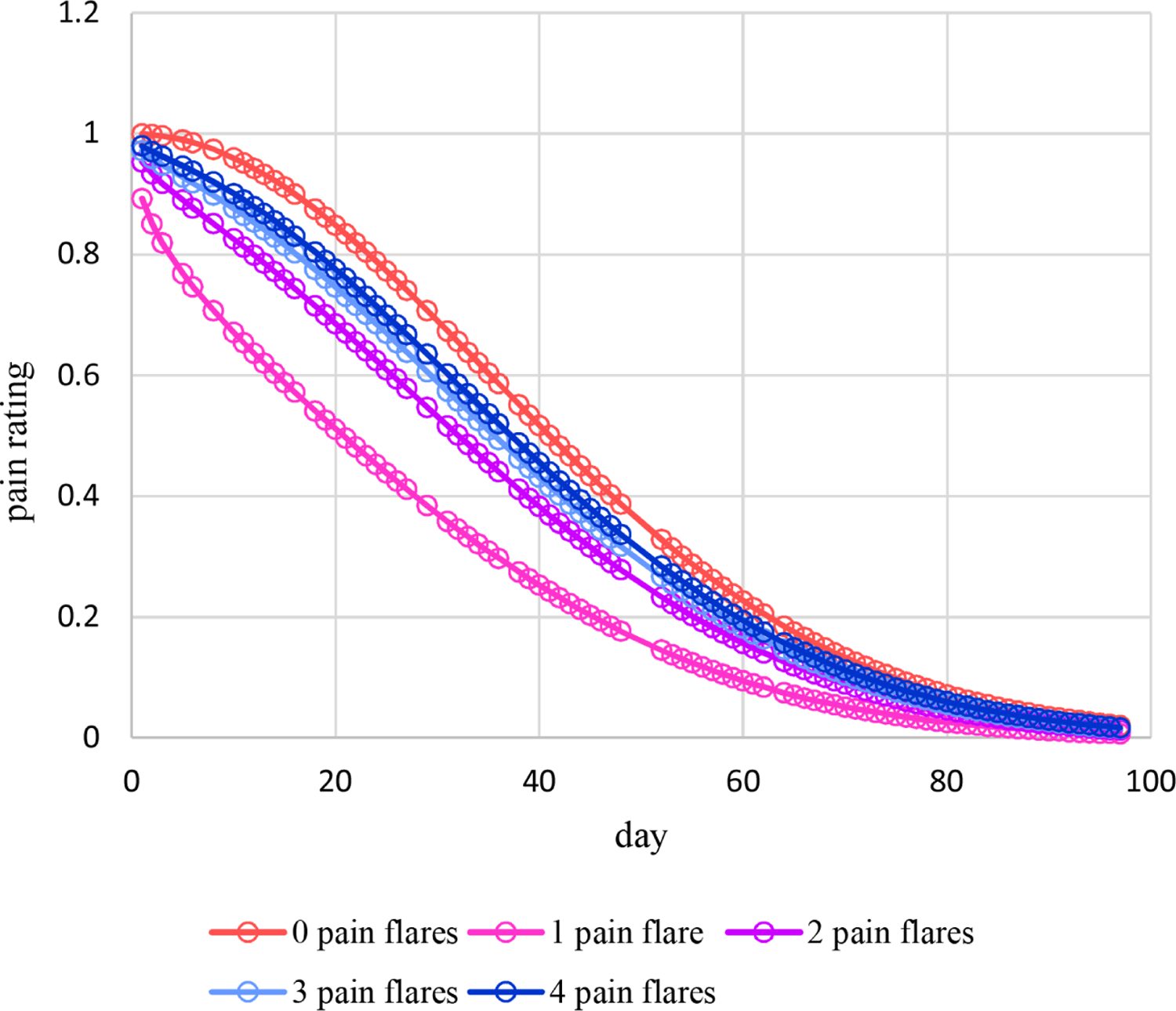
Estimated dispersions for pain ratings over time moderated by the number of pain flares for Cancer Patient 1.

**Figure 11.**
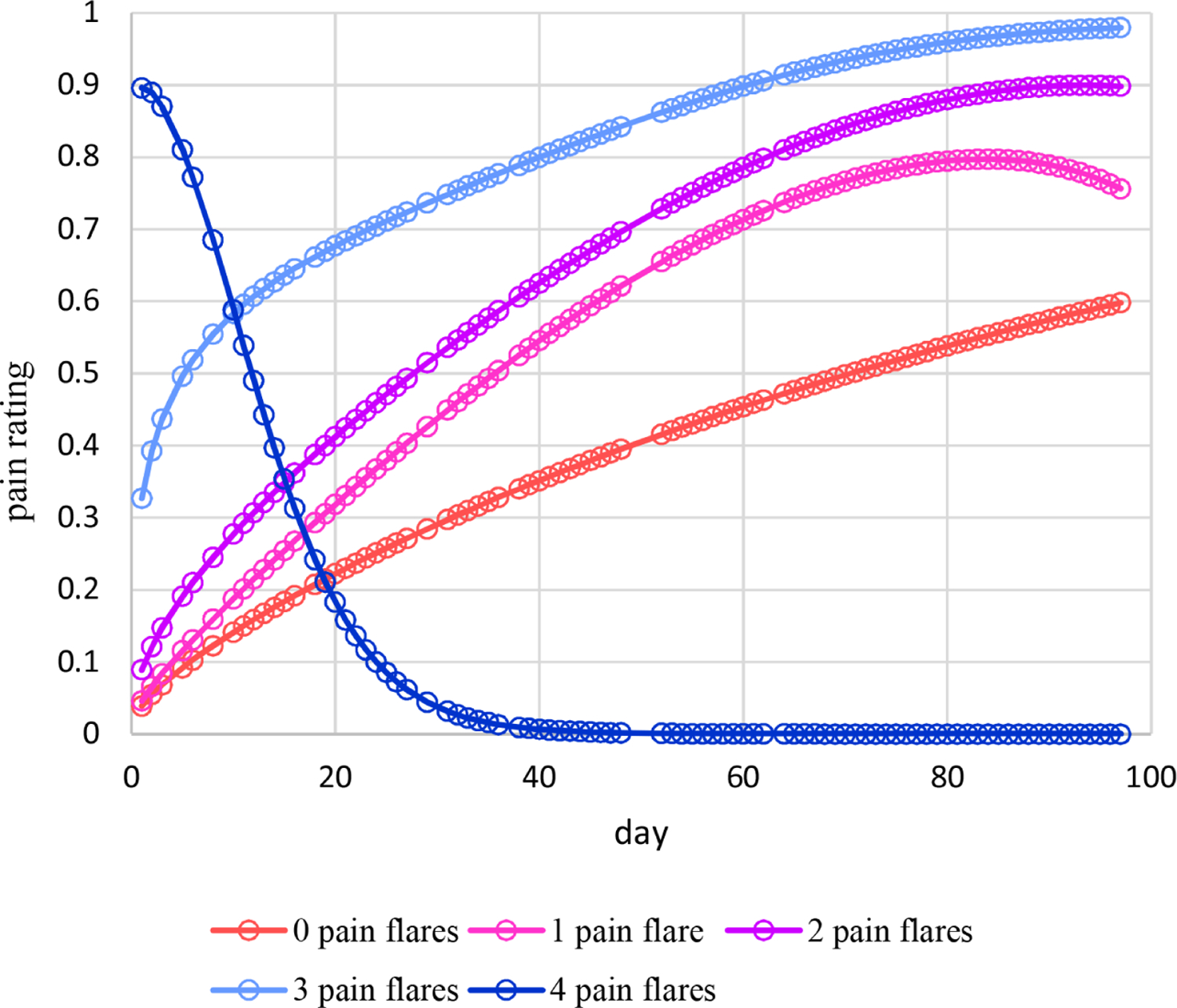
Estimated probabilities of a high pain rating of 6 or more over time moderated by the number of pain flares for Cancer Patient 1.

Estimated means under this moderation model are plotted in Figure 9. For 0 – 3 pain flares, estimated means increase nonlinearly over time with some mild decreases late in time in some cases and follow somewhat different patterns. On the other hand, estimated means for 4 pain flares decrease nonlinearly over time. Figure 10 displays the plot of estimated dispersions for the moderation model. Estimated dispersions decrease nonlinearly over time following somewhat different patterns at increasingly higher levels for 1 – 4 pain flares, but at the highest level at 0 pain flares. Plots for estimated probabilities are not provided because that requires two plots similar to Figure 4 and Figure 5 for each the five observed numbers of pain flares. However, Figure 11 provides the plot for estimated probabilities of a high pain rating of 6 or more. Similar to the means of Figure 9, estimated probabilities for 0 – 3 pain flares increase nonlinearly over time with some mild decreases late in time in some cases and following somewhat different patterns while estimated probabilities for 4 pain flares decrease nonlinearly over time from a high level at day 1 to essentially zero from around day 40 and later.

## 4. Summary

Formulations are provided for methods to use in regression modeling of individual-patient longitudinal discrete outcomes allowing for nonlinearity in predictors for probabilities and dispersions for such outcomes along with temporal correlation using spatial autoregression order 1. Three approaches are considered for modeling probabilities of outcome values. The multinomial approach is based on generalized logits with separate intercept and slope parameters for modeling probabilities for outcome values. The ordinal approach is based on cumulative logits with separate intercept parameters and the same slope parameter for modeling cumulative probabilities for outcome values. The censored Poisson approach is based on the log link function with the same intercept and slope parameters for modeling standard Poisson probabilities for all but the largest outcome value, whose value is set so that the probabilities sum to one.

Extended linear mixed modeling is used to estimate model parameters for the three probability types. A likelihood-like function *L* is defined using the multivariate normal density evaluated using residuals and covariances for discrete outcomes. The function *L* is maximized by solving estimating equations corresponding to setting the gradient vector equal to zero. Formulations are provided for computing gradient vectors and Hessian matrices for use in estimating models of each probability type. The function *L* is used to compute likelihood-like cross-validation (LCV) scores for comparing alternative models. These LCV scores are used to control an adaptive modeling process for heuristic search through power transforms of available predictors of outcome probabilities and dispersions.

These methods are used in example adaptive analyses of the longitudinal individual-patient cancer pain ratings of Figure 1. Table 1 contains results for generated models of these pain ratings in time using each of the three probability types. The censored Poisson approach is preferable over the other two approaches for modeling these data because the associated model has a competitive LCV score, is more parsimonious based on fewer parameters (three compared to nine for each of the other two approaches), and is computed in much less time. This is likely to hold for modeling of other longitudinal discrete outcomes collected for individual patients, not just discrete outcomes based on pain ratings, and even of longitudinal discrete outcomes for multiple patients. The censored Poisson model for the example data has estimated probabilities that are nonlinear in time (Figures 4–6) generating associated means (Figure 2) and dispersions (Figure 3) that are also nonlinear in time.

Models are also generated assessing the additive effect of the number of pain flares on means and dispersions (Figure 7 and Figure 8) as well as moderation of the effect of time by the number of pain flares (Figures 9–11). There is an additive effect compared to the model based on only time, but a more substantive moderation effect. These models demonstrate the need to account for nonlinear additive and moderation effects for individual-patient longitudinal discrete outcomes.

Future research is needed to assess the use of ELMM for modeling correlated discrete outcomes for multiple patients in combination. Future research is also needed to compare ELMM to generalized linear mixed modeling.

## Figures and Tables

**Table 1.** Comparison of Probability Types for Analyzing Daily Pain Ratings of Cancer Patient 1^a^.

Probability Type	Model Transforms^b^		5-Fold LCV Score	Number of Parameters	Clock Time (Minutes)
	Probabilities	Dispersions			
multinomial	time^1.7999^	time^2.5009^	0.23083	9	42.7
ordinal	time^1.3089^	time^5.1^	0.22635	9	25.9
censored Poisson	time^0.2^, time^−1^	time^7.8^	0.22935	3	5.2

LCV—likelihood cross-validation.

a Computed using adaptive extended linear mixed modeling and spatial autoregressive order 1 correlations.

b All models have zero intercept terms.
